# Prevalence and triggers of allergic rhinitis in the United Arab Emirates

**DOI:** 10.1186/1939-4551-7-19

**Published:** 2014-08-01

**Authors:** Bassam Mahboub, Suleiman Al-Hammadi, Vijayshree P Prakash, Nabil Sulaiman, Michael S Blaiss, Abdulla Al Redha, Deepa M Vats

**Affiliations:** 1 Department of Pulmonary Medicine, Rashid Hospital, Dubai, UAE; 2 Department of Pediatrics, College of Medicine and Health Sciences, UAE University, Al-Ain, Dubai, UAE; 3 Department of Family & Community Medicine, Sharjah University, Sharjah, UAE; 4 Department of Pediatrics and Medicine, University of Tennessee Health Science Centre, Memphis, Tennessee; 5 Department of Otorhinolaryngology, Al-Qassimi Hospital, Sharjah, Dubai, UAE

**Keywords:** Allergens, Nasal mucosa, Pollen, Air pollution, Fine dust, Constructions, Gulf region

## Abstract

**Background and objectives:**

Allergic rhinitis is a morbid condition that is frequently overlooked by patients and physicians. This type of atopy has not been adequately investigated in the United Arab Emirates.

**Methods:**

This cross-sectional, population-based observational study was conducted in the seven Emirates (Abu Dhabi, Dubai, Sharjah, Ajman, Umm Al-Quwain, Ras Al-Khaimah, and Fujairah). It used the European Community Respiratory Health Survey (ECRHS II) to screen for allergic rhinitis in people living in this region.

**Results:**

Symptoms of allergic rhinitis were present in 85 (7%) of the 1,229 study population. Only 33 (39%) patients received treatment. Seventy-six (89%) patients had asthma. Thirty-seven (44%) patients were poly-sensitized. Symptoms were aggravated by dust (59%), grass/pollens (44%) and proximity to animals (21%). Winter was the peak season (37%), followed by spring (30%), autumn (18%) and summer (15%). Grass/pollen allergies were clustered in the winter, spring and summer (*p* ≤ 0.001). Dust was non-seasonal (*p* ≥ 0.121) and animal allergy was worse in the winter (*p* = 0.024) and spring (*p* = 0.044). Spring symptoms were less common in people living in the inner city (*p* = 0.003).

**Conclusions:**

At least 7% of the studied population had allergic rhinitis. Most (71%) of these patients had environmental triggers and remained untreated. Allergic rhinitis awareness and measures to control allergens and dust are needed. The impact of preventing allergic rhinitis on other common atopies in the region deserves future studies.

## Introduction

Allergic rhinitis is an IgE-mediated inflammation of the nasal mucosa that is frequently triggered by inhaled allergens [1]. The symptoms are often ignored by patients and physicians, and most affected individuals do not report their complaints or seek treatments [2]. Clinical findings include recurrent sneezing, rhinorrhea, nasal congestion (stuffy nose), nasal/palatal itching, and itchy/watery eyes. These manifestations are associated with daytime somnolence, disturbed sleep, reduced activity and absenteeism from school or work [3]. Patients may also have other atopies (e.g., sinusitis, conjunctivitis, asthma, and atopic dermatitis) [4] or demonstrate a poor response to atopy treatment [1].

Prevalence of allergic rhinitis has been recently estimated by the World health organization (http://www.who.int/gard/publications/GARDGMreport2013.pdf). A reported prevalence of self-reported seasonal and perennial nasal symptoms in the United States is 30% [5], in Europe 20% [6,7], in different parts of the Middle East 9% [8], and in Al-Ain City (Abu Dhabi) 36% [9]. This study used personal interviews and bilingual English and Arabic version of the European Community Respiratory Health Survey (ECRHS II) to investigate the prevalence and triggers of allergic rhinitis in the United Arab Emirates (UAE).

## Method

This cross-sectional, population-based observational study enrolled adults from regions across the entire UAE. The participants (n = 1,229; 20% citizens and 80% expatriates) were random representatives of the inner cities, suburbs, small towns, villages, and farms. The study sample size was calculated based on the “UAE Census Estimation - 2009”, as previously described [10]. There were 455 (37%) females and 774 (63%) males.

ECRHS II was used to identify individuals with nasal or eye symptoms that were consistent with allergic rhinitis. A face-to-face community survey was conducted across the seven Emirates (Abu Dhabi, Dubai, Sharjah, Ajman, Umm Al-Quwain, Ras Al-Khaimah, and Fujairah) between January 2010 and March 2010. All participants were informed about the voluntary nature of the study and a signed written informed consent was obtained from each participant. There were no exclusion criteria. The research protocol was approved by the Clinical Research Ethics Authority of Dubai.

The ECRHS II screener questionnaire was translated to Arabic and the interviews were conducted using a side by side bilingual (English/Arabic) version (Additional file 1). Patient reports of environmental triggers and use of nasal allergy medications were noted. All interviewers were trained prior to the survey. Each interviewer conducted two pilot interviews and reviewed the completed questionnaires with a supervisor to identify and resolve inconsistencies. All materials were piloted as per standard quality control procedures. Each interviewer interviewed at least ten subjects and the process was witnessed by an experienced person who could clarify doubtful points. Completed questionnaires were checked regionally and again centrally for accuracy.

Participants who answered “yes” to at least one of the four designated questions (Question 1, 4, 5, or 7) in the screener questionnaire were invited to answer the modified, bilingual version (English and Arabic) of ECRHS II main questionnaire (Additional file 2). The modification included local/regional nuances, such as shisha smoking and use of trade/brand names of anti-allergic drugs or nasal sprays. Participants who also answered “yes” to Question 7 were considered having allergic rhinitis. The main questionnaire was administered immediately after the screening, or follow-up appointments were arranged between the interviewer and respondent to complete the 86 questions of the questionnaire.

Question one was: Have you had wheezing or whistling in your chest at any time in the last 12 months? Question four was: Have you been woken by an attack of coughing at any time in the last 12 months? Question five was: Have you had an attack of asthma in the last 12 months? Question seven was: Do you have any nasal allergies including hay fever?

The Statistical Package for Social Sciences (SPSS) software version 19.0 for Windows was used. Logistic regression analysis was used to identify independent predictors of triggers (dust, grass/pollens, and animal proximity) and seasons of symptoms. *P* < 0.05 was considered significant.

## Results

One hundred eighty-eight (15%) of the 1,229 participants answered “yes” to Question 1, 4, 5, or 7, in the screener questionnaire (see above). Of those, 85 (7%) patients answered “yes” to Question 7, and were diagnosed as having allergic rhinitis. Twenty-two (27%) patients were ≤19 years of age, 46 (55%) were 20–44 years of age, and 15 (18%) were ≥45 years of age; two patients did not report their ages. Fifty-eight percent of the patients lived in inner cities, 25% in suburbs, 8% in small towns, 7% in rural areas and 2% in farms.

Seventy-six (89%) of the 85 patients had asthma. Sixty-one percent of the patients did not receive any treatment, 13% received steroid nasal spray, 5% received oral antihistamines and 14% received steroid nasal spray plus antihistamines.

Thirty-seven (44%) patients were poly-sensitized. House-hold dust was a common trigger; 50 (59%) patients reported worsening of symptoms in dusty areas of their homes or near dusty pillows, rugs or duvets. Grass/pollens were triggers in 37 (44%) patients and proximity to animals in 18 (21%) patients (Table 1). Twelve patients (14%) reported no worsening of the nasal symptoms on exposure to dust, grass/pollens or animals; however, their asthma worsened when exposed to these allergens/irritants. Nine (11%) patients reported no worsening of either nasal or asthma symptoms following dust, grass/pollen or animal exposure.

**Table 1:** Triggers and seasonality of the allergic rhinitis

	**Males**	**Females**	**Total**
	**N = 774**	**N = 455**	**N = 1229**
	**n (%)**	**n (%)**	**n (%)**
**Triggers**			
Dust only	13 (29)	9 (23)	22 (26)
None specified	13 (29)	8 (20)	21 (25)
Dust + grass/pollens	7 (16)	10 (25)	17 (20)
Dust + grass/pollens + animals	4 (8)	5 (13)	9 (11)
Grass/pollens only	3 (7)	4 (10)	7 (8)
Grass/pollens + animals	2 (4)	2 (5)	4 (4)
Animals only	1 (2)	2 (5)	3 (4)
Dust + animals	2 (4)	0	2 (2)
**Seasonality**			
Winter	15 (37)	14 (38)	29 (37)
Spring	13 (32)	10 (27)	23 (30)
Summer	6 (15)	6 (16)	12 (15)
Autumn	7 (17)	7 (19)	14 (18)

Values are number (%) of patients with available data. Seasonality was not reported in seven patients.

Forty-eight percent of the patients lived near industrial areas or constructions; 74% reported constant/frequent passing of vehicles near their homes. Forty-six percent of patients made efforts to minimize exposure to dust and dust-mites by changing carpets and using anti-mite sprays or covers.

Thirty-seven percent of patients reported worsening of symptoms in the winter (November-February) and 30% reported worsening of symptoms in the spring (March-May). Summer and autumn were less frequent seasons, since many people traveled outside the country in the summer and returned in early autumn (Table 1). Three (4%) patients reported perennial symptoms and four (5%) reported no relation to seasons.

Grass/pollen allergies were clustered in the winter, spring and summer (*p* ≤0.001), Table 2. Dust was non-seasonal (*p* ≥0.121) and animal allergy was in the winter (*p* = 0.024) and spring (*p* = 0.044), Table 2. Living in the inner city was not a predictor of any trigger (*p* ≥0.374), and spring symptoms were less common in people living in the inner city (*p* = 0.003), Table 3.

**Table 2:** **Simple logistic regression of triggers of allergic rhinitis *vs. *** **season**

**Variable**	**Trigger**	**OR**	**95% CI for OR**	***P*-value**
Winter	Dust			
Yes	22 (76)	2.2	0.8 – 6.0	0.121
No	33 (59)			
Winter	Grass/pollens			
Yes	26 (90)	28.7	7.5 – 110.2	<0.001
No	13 (23)			
Winter	Animal proximity			
Yes	11 (38)	3.2	1.1 – 9.0	0.024
No	9 (16)			
Spring	Dust			
Yes	15 (65)	1.0	0.4 – 2.8	0.952
No	40 (65)			
Spring	Grass/pollens			
Yes	19 (83)	10.0	3.0 – 33.2	<0.001
No	20 (32)			
Spring	Animal proximity			
Yes	9 (39)	3.0	1.0 – 8.6	0.044
No	11 (18)			
Summer	Dust			
Yes	10 (83)	3.2	0.7 – 15.6	0.137
No	44 (61)			
Summer	Grass/pollens			
Yes	11 (92)	18.3	2.2 – 150.0	<0.001
No	27 (38)			
Summer	Animal proximity			
Yes	4 (33)	1.8	0.5 – 6.6	0.403
No	16 (22)			
Autumn	Dust			
Yes	10 (71)	1.5	0.4 – 5.2	0.541
No	44 (63)			
Autumn	Grass/pollens			
Yes	10 (71)	3.8	1.0 – 13.1	0.039
No	28 (40)			
Autumn	Animal proximity			
Yes	4 (29)	1.4	0.4 – 4.9	0.647
No	16 (23)			

Values are n (%). OR, odds ratio; CI, confidence interval.

**Table 3:** **Simple logistic regression of triggers of allergic rhinitis *vs. *** **living area**

**Variable**	**Trigger or season**	**OR**	**95% CI for OR**	***P*-value**
Inner city	Dust			
Yes	32 (67)	1.2	0.5 – 3.0	0.667
No	23 (62)			
Inner city	Grass/pollens			
Yes	20 (42)	0.7	0.3 – 1.6	0.374
No	19 (51)			
Inner city	Animal proximity			
Yes	13 (27)	1.6	0.6 – 4.5	0.379
No	7 (19)			
Inner city	Winter			
Yes	33 (69)	1.3	0.5 – 3.3	0.525
No	23 (62)			
Inner city	Spring			
Yes	29 (60)	0.2	0.1 – 0.6	0.003
No	33 (81)			
Inner city	Summer			
Yes	43 (92)	3.0	0.8 – 10.8	0.088
No	29 (78)			
Inner city	Autumn			
Yes	39 (83)	0.9	0.3 – 3.0	0.922
No	31 (84)			

Values are n (%). Living area was inner city *vs.* other areas. OR, odds ratio; CI, confidence interval.

## Discussion

Widespread variations in the prevalence of allergic rhinitis have been reported worldwide [11,12]. The overall prevalence across Europe (Belgium, France, Germany, Italy, Spain and Britain) is about 23%, (lowest in Italy and highest in Belgium) [7]. Using Score for Allergic Rhinitis (SFAR) questionnaire, the prevalence in Turkey is 30% (21% in Southeast Anatolia and 36% in Marmara) [13].

The prevalence of physician-diagnosed allergic rhinitis in five Middle East countries (Egypt, Iran, Lebanon, Saudi Arabia, and UAE) is 9%, with dust being the main trigger [8]. These results are similar to our study, showing a prevalence of 7% and dust being a common trigger (Table 1). Mesquite (leguminous tree of the *Prosopis* genus) is the most common allergen in Turkey [14].

The prevalence of allergic rhinitis in school children from Saudi Arabia is 24%, Turkey 16%, Kuwait 41%, Lebanon 45%, and Al-Ain city (Abu Dhabi) 36% [9,15-18]. Being an inland desert oasis, Al-Ain city is rich in plantation type plants. It has the highest number of date palms and gardens, which explain the frequent plant-induced allergic rhinitis [9]. Dubai, Umm Al-Quwain and Ajman are desert areas with numerous constructions that produce fine dust. Fujairah contains mountains that are rich in plant life. Ras Al-Khaimah has cement factories and ceramic industries. These environmental variables undoubtedly attribute to the natural history of allergic rhinitis in the region.

Construction produces numerous amounts of fine dust in the air, which is a major source of airway irritation. Consistently, dust is shown here to be a main airway irritant (Tables 1 and 2). Being in a dusty room and exposure to a dusty rug/pillow aggravated the symptoms in 59% of the studied patients (Table 1). Dust mites were a main trigger in patients from Israel, Iran, and Jordan [19-21]. Moreover, grass/pollens were found to be a common allergen in patients from Iran and Turkey [22,23].

Many (44%) patients were poly-sensitized. Thirty-seven (58%) of the patients with a specific trigger had grass/pollens allergy. Dust alone was a trigger in only 22 (26%) patients (Table 1). Seasons and living in the inner city were not significant predictors of dust-induced symptoms (Tables 2 and 3). In contrast, grass/pollens were main triggers for people who identified a season for their symptoms (Table 2).

A high prevalence of allergies in urban (compared to rural) areas was reported in Germany, Poland, and Turkey [24-26]. Our inner cities have unique living conditions including high humidity, use of air conditioning, frequent house molds and traffic pollution. Nevertheless, spring allergies were less frequent in people living in the inner city (Table 3, *p* = 0.003).

Only 46% of the patients made efforts to minimize exposure to dust and dust-mites; only 39% received treatment. Other studies have shown low prevalences of allergic rhinitis treatment (12% in USA, 47% in Europe, and 53% in Middle East) [7,9,12]. This practice of not treating patients reflects inadequate allergic rhinitis awareness on the part of doctors and patients. Since allergic rhinitis may be a precursor to (risk factor for) asthma, more emphasis on this disorder is needed.

### Study limitations

This study employed the standard ECRHS II for identifying patients with allergic rhinitis. The questionnaire did not address severity of symptoms and did not include a physician diagnosis. Furthermore, this survey was performed in a population with little awareness about allergic rhinitis. Thus, the symptoms might have been underreported. The number of patients was small for sub-analysis by individual Emirates. This important limitation should be addressed in a future study. Follow-up studies should include physician documentations of the allergic symptoms and patients’ response to interventions.

## Conclusions

Prevalence of allergic rhinitis in the UAE is at least 7%. Dust and grass/pollens are common triggers. Improving public awareness and implementing preventive measures are necessary for controlling allergic rhinitis in the region.

## Competing interests

The authors declare that they have no competing interests.

## Authors’ contributions

BM have made substantial contributions to conception and design, acquisition of data, analysis and interpretation of data and drafting the manuscript and revising it critically for important intellectual content. SAH have made substantial contributions to analysis and interpretation of data, drafting the manuscript, critical revision for important intellectual content, statistical analysis and rewriting the manuscript according to the reviewers' opinions. VPP, AAR and DMV have made substantial contributions to statistical analysis, acquisition of data, and have given their final approval of the version to be published. NS have made substantial contributions to analysis and interpretation of data, and have given final approval of the version to be published. MSB have made critical revision for important intellectual content, drafting the manuscript, and have given final approval of the version to be published. All authors read and approved the final manuscript.

## Supplementary Material

Additional file 1 Screener Questionnaire. Click here for file

Additional file 2 Main Questionnaire. Click here for file
