# Supplementary material for: Prevalence and triggers of allergic rhinitis in the United Arab Emirates
Source: World Allergy Organ J. 2014 Aug 1;7(1):19. doi: 10.1186/1939-4551-7-19 (PMC4118622; doi:10.1186/1939-4551-7-19)
Supplement: Additional file 1 — Screener Questionnaire. [file 1939-4551-7-19-S1.pdf]

Interviewer Name		اسم الباحث
Respondent Name		اسم المجيب
Telephone number		رقم الهاتف
Email address/Fax		عنوان البريد الإلكتروني/ الفاكس
Date of Interview		تاريخ المقابلة
Phone No (Mobile)		رقم الهاتف (المحمول)
Residence No		رقم الإقامة
Office No		رقم المكتب

Hello. My name is _________ and I am conducting a survey on behalf of Datamonitor, a global consulting firm. We are currently conducting a study to understand how common Asthma and chronic respiratory diseases are among the UAE population. Would you be interested to participate in this health and lifestyle survey?

مرحباً. أنا اسمي _________ وأجري دراسة لصالح شركة داتا مونيتر (Datamonitor)، شركة استشارات عالمية. نحن نجري دراسة لكي نعرف مدى انتشار أزمة الربو و الأمراض التنفسية المزمنة بين سكان الإمارات العربية المتحدة. هل لديك الرغبة فى المشاركة فى هذه الدراسة التى تتناول الصحة وأسلوب المعيشة؟

You would be required to answer a screening questionnaire which will only take about 3 minutes of your time. If you then qualify for the main questionnaire, and answer all of its 86 questions, you will be given a free consultation and lung test by a leading pulmonologist in a private hospital in Dubai.

سيتطلب منك الأمر الإجابة على استمارة انتقاء والتي سوف تستغرق حوالي 3 دقائق فقط من وقتك. إذا تم تأهلك للاستمارة الرئيسية، واستطعت الإجابة على 86 سؤال الموجودين بها، سوف تحصل على استشارة مجانية و اختبار للرئة من قبل أخصائي صدر ماهر فى مستشفى خاصة بدبي.

The data gathered from this study will be used solely for scientific purposes, NOT for any marketing or sales purposes. Your identity and contact information will be held strictly confidential and will only be used by the pulmonologist’s secretary to arrange an appointment with you.

سوف يتم استخدام البيانات التى تم تجميعها من أجل أغراض علمية فقط، وليس من أجل أى أغراض تسويقية أو أغراض خاصة بالمبيعات. سيتم التعامل مع هويتك و بيانات الإتصال الخاصة بك بسرية تامة و سيقوم سكرتير أخصائي الصدر فقط بالإطلاع عليها لكى يستطيع تحديد ميعاد معك.

Screener Questionnaire | **استمارة انتقاء**

1. Have you had wheezing or whistling in your chest at any time in the last 12 months?

1 . هل شعرت بأزيز أو صفير فى صدرك فى أى وقت خلال الإثنى عشر شهراً الماضية؟

Yes (Go to Q 1.1)	1	نعم (اذهب إلى Q 1.1)
No (Go to Q2)	2	لا (اذهب إلى Q2)

1.1. Have you been at all breathless when the wheezing noise was present?

1.1. هل واجهت صعوبة تامة فى التنفس عندما شعرت بالأزيز؟

Yes	1	نعم
No	2	لا

1.2. Have you had this wheezing or whistling when you did not have a cold?

1.2. هل شعرت بهذا الأزيز أو الصفير وأنت لا تعاني من إصابتك بالبرد؟

Yes	1	نعم
No	2	لا

2. Have you woken up with a feeling of tightness in your chest at any time in the last 12 months?

2. هل استيقظت من النوم و أنت تشعر بضيق فى صدرك فى أى وقت خلال الإثنى عشر شهراً الماضية؟

Yes	1	نعم
No	2	لا

3. Have you been woken by an attack of shortness of breath at any time in the last 12 months?

3. هل استيقظت من النوم بسبب نوبة ضيق تنفس فى أى وقت خلال الإثنى عشر شهراً الماضية؟

Yes	1	نعم
No	2	لا

4. Have you been woken by an attack of coughing at any time in the last 12 months?

4. هل استيقظت من النوم بسبب نوبة من السعال في أي وقت خلال الإثنى عشر شهراً الماضية؟

Yes	1	نعم
No	2	لا

5. Have you had an attack of asthma in the last 12 months?

5. هل أصبت بأزمة ربو خلال الإثنى عشر شهراً الماضية؟

Yes	1	نعم
No	2	لا

6. Are you currently taking any medicine (including inhalers, aerosols or tablets) for asthma?

6. هل تتناول في الوقت الحالي أي دواء (بما في ذلك أدوية الاستنشاق أو البخاخات أو الأقراص) لعلاج أزمة الربو؟

Yes	1	نعم
No	2	لا

7. Do you have any nasal allergies including hay fever?

7. هل تعاني من أية حساسية في الأنف تشمل حمى القش؟

Yes	1	نعم
No	2	لا

8. What is your date of birth? (DAY MONTH YEAR)

8. ما هو تاريخ ميلادك؟ (اليوم الشهر السنة)

____

9. What is today's date? Interviewer will record (DAY MONTH YEAR)

9. ما هو تاريخ اليوم؟ سيسجل الباحث (اليوم الشهر السنة)

____

10. Are you male or female?

10. هل أنت ذكر أم أنثى؟

Male	1	ذكر
Female	2	أنثى

THANK YOU FOR YOUR HELP

نشكرك على مساعدتك

Interviewer to note:

على الباحث أن يسجل:

Criteria for qualification for Main Questionnaire:

1. Answer YES to Q1 about wheezing/ whistling in the chest; or
2. Answer YES to Q4 about being woken up by an attack of coughing in the last 12 months; or
3. Answer YES to Q5 about having had an attack of asthma in the last 12 months; or
4. Answer YES to Q7 about having nasal allergies including hay fever.

معايير التأهيل الخاصة بالاستمارة الرئيسية:

1. اجاب نعم عن السؤال Q1 فيما يخص الأزيز/ الصفير في الصدر; أو
2. اجاب نعم عن السؤال Q4 فيما يخص السعال باستمرار لأكثر من 3 أسابيع في أي وقت من حياتك; أو
3. اجاب نعم عن السؤال Q5 فيما يخص الإصابة بأزمة الربو خلال الـ 12 شهراً الماضية; أو
4. اجاب نعم عن السؤال Q7 فيما يخص الإصابة بحساسية في الأنف تشمل حمى القش.
