# Supplementary material for: Prevalence and triggers of allergic rhinitis in the United Arab Emirates
Source: World Allergy Organ J. 2014 Aug 1;7(1):19. doi: 10.1186/1939-4551-7-19 (PMC4118622; doi:10.1186/1939-4551-7-19)
Supplement: Additional file 2 — Main Questionnaire. [file 1939-4551-7-19-S2.pdf]

## Main Questionnaire

I AM GOING TO ASK YOU SOME QUESTIONS. AT FIRST THESE WILL BE MOSTLY ABOUT YOUR BREATHING. WHEREVER POSSIBLE I WOULD LIKE YOU TO ANSWER 'YES' OR 'NO'.

سأطرح عليك بعض الأسئلة. ستكون هذه الأسئلة فى المقام الأول عن عملية التنفس. أريدك أن تقوم بالإجابة إما بـ "نعم" أو "لا".

1. Have you had wheezing or whistling in your chest at any time in the last 12 months?

1. هل شعرت بأزيز أو صفير داخل صدرك خلال الـ 12 شهراً الماضية؟

| | | |
|---|---|---|
| Yes (Goto Q1.1) | 1 | نعم (انتقل إلى Q1.1) |
| No (Go to Q2) | 2 | لا (انتقل إلى Q2) |

1.1 Have you been at all breathless when the wheezing noise was present?

1.1 هل شعرت من قبل بأنك لا تتنفس عند وجود صوت الأزيز هذا؟

| | | |
|---|---|---|
| Yes | 1 | نعم |
| No | 2 | لا |

1.2. Have you had this wheezing or whistling when you did not have a cold?

1.2 هل شعرت بهذا الصفير أو الأزيز من قبل في وقت لم تكن تعاني فيه من البرد؟

| | | |
|---|---|---|
| Yes | 1 | نعم |
| No | 2 | لا |

2. Have you woken up with a feeling of tightness in your chest at any time in the last 12 months?

2. هل استيقظت وأنت تشعر بضيق فى صدرك فى أي وقت خلال الـ 12 شهراً الماضية؟

| | | |
|---|---|---|
| Yes | 1 | نعم |
| No | 2 | لا |

3. Have you had an attack of shortness of breath that came on during the day when you were at rest at any time in the last 12 months?

3. هل تعرضت لأي نوبة ضيق فى التنفس خلال يوم راحة فى أى وقت خلال الـ 12 شهراً الماضية؟

| | | |
|---|---|---|
| Yes | 1 | نعم |
| No | 2 | لا |

4. Have you had an attack of shortness of breath that came on following strenuous activity at any time in the last 12 months?

4. هل تعرضت لأية نوبة ضيق فى التنفس بعد قيامك بعمل نشاط عنيف فى أى وقت خلال الـ 12 شهراً الماضية؟

| | | |
|---|---|---|
| Yes | 1 | نعم |
| No | 2 | لا |

5. Have you been woken by an attack of shortness of breath at any time in the last 12 months?

5. هل استيقظت بفعل أية نوبة ضيق فى التنفس فى أى وقت خلال الـ 12 شهراً الماضية؟

| | | |
|---|---|---|
| Yes (Goto Q5.1) | 1 | نعم (انتقل إلى Q5.1) |
| No (Go to Q6) | 2 | لا (انتقل إلى Q6) |

5.1 Have you been woken by an attack of shortness of breath in the last 3 months?

5.1 هل استيقظت بفعل أية نوبة ضيق فى التنفس فى أى وقت خلال الـ 3 أشهر الماضية؟

| | | |
|---|---|---|
| Yes (Goto Q5.1.1 ) | 1 | نعم (انتقل إلى Q5.1.1) |
| No (Go to Q6) | 2 | لا (انتقل إلى Q6) |

5.1.1 On average have you been woken by an attack of shortness of breath at least once a week in the last 3 months?

5.1.1 فى المتوسط، هل استيقظت بفعل أية نوبة ضيق فى التنفس على الأقل مرة واحدة فى الأسبوع خلال الـ 3 أشهر الماضية؟

| | | |
|---|---|---|
| Yes (Go to Q5.1.1.1 ) | 1 | نعم (انتقل إلى Q5.1.1.1) |
| No (Go to Q6) | 2 | لا (انتقل إلى Q6) |

5.1.1.1 How many times a week on average have you been woken by shortness of breath in the last 3 months?

5.1.1.1 فى المتوسط، كم مرة فى الأسبوع استيقظت بفعل نوبة ضيق فى التنفس خلال الـ 3 أشهر الماضية؟

| | | |
|---|---|---|
| Average number of times a week | | متوسط عدد المرات فى الأسبوع |

6. Have you been woken by an attack of coughing at any time in the last 12

6. هل استيقظت بفعل نوبة سعال فى أى وقت خلال الـ 12 شهراً الماضية؟

| | | |
|---|---|---|
| Yes | 1 | نعم |
| No | 2 | لا |

7. Do you usually cough first thing in the morning in the winter? [IF DOUBTFUL, USE QUESTION 8.1 TO CONFIRM]

7. هل دائماً ما تسعل أول شىء عندما تستيقظ صباحاً فى الشتاء؟
[اذا كان غير متأكداً، اطرح عليه السؤال 8.1 للتأكيد]

| | | |
|---|---|---|
| Yes (Go to Q8) | 1 | نعم |
| No ( Go to Q8) | 2 | لا |
| Don't know/not sure( Go to Q8.1) | 3 | |

8. Do you usually cough during the day, or at night, in the winter?

8. هل دائماً ما تسعل خلال النهار، أم فى الليل، أثناء الشتاء؟

| | | |
|---|---|---|
| Yes (Go to Q 8.1 ) | 1 | نعم (انتقل إلى Q8.1) |
| No (Go to Q 9) | 2 | لا (انتقل إلى Q9) |

8.1 Do you cough first thing in the morning for as much as three months each year?

8.1 هل تستيقظ في الصباح أول شيء على سعال بمعدل يقرب من ثلاثة أشهر كل عام؟

| | | |
|---|---|---|
| Yes | 1 | نعم |
| No | 2 | لا |

9. Do you usually bring up any phlegm from your chest first thing in the morning in the winter?

9. هل دائماً ما تُخرج أى بلغم من صدرك أول شيء صباحاً فى الشتاء؟

| | | |
|---|---|---|
| Yes (Go to Q10) | 1 | نعم (انتقل إلى Q10) |
| No (Go to Q10) | 2 | لا (انتقل إلى Q10) |
| Don't know/not sure ( Go to Q10.1) | | لا أعرف (انتقل إلى Q10.1) |

| | |
|---|---|
| [IF DOUBTFUL USE QUESTION 10.1 TO CONFIRM] | [اذا كان غير متأكداً، اطرح عليه السؤال 10.1 للتأكيد] |

10. Do you usually bring up any phlegm from your chest during the day, or at night, in the winter?

10. هل دائماً ما تُخرج أى بلغم من صدرك خلال النهار، أو فى الليل، أثناء الشتاء؟

| | | |
|---|---|---|
| Yes (Go to Q 10.1 ) | 1 | نعم (انتقل إلى Q 10.1) |
| No (Go to Q 11) | 2 | لا (انتقل إلى Q 11) |

10.1 Do you bring phlegm up from your chest first thing in the morning for as much as three months each year?

10.1 هل تُخرج بلغم من صدرك أول شيء عندما تستيقظ في الصباح بمعدل يقرب من ثلاثة أشهر كل عام؟

| | | |
|---|---|---|
| Yes | 1 | نعم |
| No | 2 | لا |

11. Do you ever have trouble with your breathing?

11. هل سبق لك وأن تعرضت لمشكلة تتعلق بالتنفس؟

| | | |
|---|---|---|
| Yes (Go to Q 11.1 ) | 1 | نعم (انتقل إلى Q 11.1) |
| No (Go to Q 12) | 2 | لا (انتقل إلى Q 12) |

11.1 Do you have this trouble (SA)

11.1 هل تعاني من هذه المشكلة (إجابة واحدة)

| | | |
|---|---|---|
| Continuously so that you're breathing is never quite right? | 1 | باستمرار، حتى أنك لم يسبق لك وأن تنفست بالشكل الصحيح؟ |
| repeatedly, but it always gets completely better? | 2 | بصورة متكررة، ولكنه دائماً ما يتحسن تماماً؟ |
| only rarely? | 3 | نادراً فقط؟ |

12. Are you disabled from walking by a condition other than heart or lung disease?

12. هل تعجز عن المشى بسبب حالة أخرى لا تتعلق بمرض فى القلب أو الرئة؟

| | | |
|---|---|---|
| Yes ( IF 'YES' STATE CONDITION ___ ________ AND GO TO Q 13 | 1 | نعم ( اذا كانت الإجابة نعم، اذكر هذه الحالة___________ ثم انتقل إلى السؤال 13 |
| No ( Go to Q12.1 ) | 2 | لا (انتقل إلى Q 12.1) |

12.1 Are you troubled by shortness of breath when hurrying on level ground or walking up a slight hill?

12.1 هل تعانى من ضيق فى التنفس عندما تمشى مُسرعاً على مستوى الأرض، أو عند صعودك إلى مكانٍ مرتفع بنسبة صغيرة؟

| | | |
|---|---|---|
| Yes (Go to Q 12.1.1 ) | 1 | نعم (انتقل إلى Q 12.1.1) |
| No (Go to Q 13) | 2 | لا (انتقل إلى Q 13) |

12.1.1 Do you get short of breath walking with other people of your own age on level ground?

12.1.1 هل تعانى من ضيق فى التنفس عندما تقوم بالتنزه مع أشخاص آخرين فى نفس عُمرك على مستوى الأرض؟

| | | |
|---|---|---|
| Yes (Go to Q 12.1.1.1 ) | 1 | نعم (انتقل إلى Q 12.1.1.1) |
| No (Go to Q 13) | 2 | لا (انتقل إلى Q 13) |

12.1.1.1 Do you have to stop for breath when walking at your own pace on level ground?

12.1.1.1 هل تضطر إلى التوقف للتنفس عندما تمشى بسرعتك المعتادة على مستوى الأرض؟

| | | |
|---|---|---|
| Yes | 1 | نعم |
| No | 2 | لا |

**FOR WOMEN ONLY - MEN GO TO Q14**

**للنساء فقط – الرجال انتقل إلى Q14 .**

Q 13 Have you ever noticed that you had respiratory symptoms (such as wheeze, tightness in your chest or shortness of breath) at a particular time of your monthly cycle?

Q13هل لاحظتِ من قبل وجود أعراض تنفسية ( مثل الأزيز، ضيق فى الصدر أو ضيق فى التنفس ) فى وقت معين من أوقات الدورة الشهرية؟

| | | |
|---|---|---|
| yes, in the week before my period | 1 | نعم خلال الأسبوع الذى يسبق الدورة |
| yes, during my period | 2 | نعم، خلال الدورة |
| yes, in the week after my period | 3 | نعم، فى الأسبوع الذى يلى الدورة |
| yes, another time of the month | 4 | نعم، فى وقتٍ آخر من الشهر |
| does not apply to me (i.e., amenorrhoeal) | 5 | لا ينطبق هذا علىّ (على سبيل المثال، تمر بفترة انقطاع الطمث) |
| No | 6 | لا |

14. Have you ever had asthma?

14. هل سبق لك وأن تعرضت لأزمة ربو؟

| | | |
|---|---|---|
| Yes (Go to Q 14.1 ) | 1 | نعم (انتقل إلى Q 14.1) |
| No (Go to Q 15) | 2 | لا (انتقل إلى Q 15) |

14.1 Was this confirmed by a doctor?

14.1 هل قام أى طبيب بالتأكيد على ذلك؟

| | | |
|---|---|---|
| Yes | 1 | نعم |
| No | 2 | لا |

14.2 How old were you when you had your first attack of asthma?

14.2 كم كان عمرك عندما تعرضت لأول أزمة ربو؟

| | | |
|---|---|---|
| Age (years) | | العمر (أعوام) |

14.3 How old were you when you had your most recent attack of asthma?

14.3  كم كان عمرك عندما تعرضت لأحدث أزمة ربو؟

| | | |
|---|---|---|
| Age (years) | | العمر (أعوام) |

14.4.1-6 Which months of the year do you usually have attacks of asthma (multiple answers)?

14.4.1-6  فى أية شهور من العام عادةً ما تتعرض لأزمات ربو (إجابات متعددة)؟

| | | |
|---|---|---|
| January / February | 1 | يناير/ فبراير |
| March / April | 2 | مارس / أبريل |
| May / June | 3 | مايو / يونيو |
| July / August | 4 | يوليو / أغسطس |
| September / October | 5 | سبتمبر / أكتوبر |
| November / December | 6 | نوفمبر / ديسمبر |

14.5 Have you had an attack of asthma in the last 12 months?

14.5 هل تعرضت لأية أزمة ربو خلال الـ 12 شهراً الماضية؟

| | | |
|---|---|---|
| Yes (Go to Q 14.6 ) | 1 | نعم (انتقل إلى Q 14.6) |
| No (Go to Q 14.8) | 2 | لا (انتقل إلى Q 14.8) |

14.6 How many attacks of asthma have you had in the last 12 months?

14.6 كم عدد نوبات أزمة الربو التى تعرضت لها خلال الـ 12 شهراً الماضية؟

| | | |
|---|---|---|
| Number of attacks | | عدد النوبات |

14.7 How many attacks of asthma have you had in the last 3 months?

14.7 كم عدد نوبات أزمة الربو التى تعرضت لها خلال الـ 3 أشهر الماضية؟

| | | |
|---|---|---|
| Number of attacks | | عدد النوبات |

14.8 How many times have you woken up because of your asthma in the last 3 months? TICK ONE BOX ONLY

14.8 كم عدد المرات التى استيقظت فيها من النوم بفعل أزمة الربو خلال الـ 3 أشهر الماضية؟ ضع علامة على مربع واحد فقط

| | | |
|---|---|---|
| every night or almost every night | 1 | كل ليلة أو تقريباً كل ليلة |
| more than once a week, but not most nights | 2 | أكثر من مرة فى الأسبوع، ولكن ليس فى معظم الليالى |
| at least twice a month, but not more than once a week | 3 | مرتان على الأقل فى الشهر، ولكن ليس أكثر من مرة فى الأسبوع |
| less than twice a month | 4 | أقل من مرتين فى الشهر |
| not at all | 5 | لم يحدث على الإطلاق |

14.9. How often have you had trouble with your breathing because of your asthma in the last 3 months? TICK ONE BOX ONLY

14.9 ما معدل حدوث مشكلة فى التنفس لك بسبب أزمة الربو التى تعانى منها، خلال الـ 3 أشهر الماضية؟ ضع علامة على مربع واحد فقط

| | | |
|---|---|---|
| continuously | 1 | باستمرار |
| about once a day | 2 | حوالى مرة فى اليوم |
| at least once a week, but less than once a day | 3 | مرة فى الأسبوع على الأقل، ولكن أقل من مرة فى اليوم |
| less than once a week | 4 | أقل من مرة فى الأسبوع |
| not at all | 5 | لم يحدث على الإطلاق |

14.10 Are you currently taking any medicines including inhalers, aerosols or tablets for asthma?

14.10 هل تتناول حالياً أى أدوية بما فى ذلك أدوية الاستنشاق، بخاخات أو أقراص لمعالجة أزمة الربو؟

| | | |
|---|---|---|
| Yes | 1 | نعم |
| No | 2 | لا |

14.11 Do you have a peak flow meter of your own?

14.11 هل لديك جهاز خاص بك لقياس قوة التنفس؟

| | | |
|---|---|---|
| Yes (Go to Q 14.11.1 ) | 1 | نعم (انتقل إلى Q 14.11.1) |
| No (Go to Q 14.12) | 2 | لا (انتقل إلى Q 14.12) |

14.11.1 How often have you used it over the last 3 months? TICK ONE BOX ONLY

14.11.1 ما معدل استخدامك له خلال الـ 3 أشهر الماضية؟ ضع علامة على مربع واحد فقط

| | | |
|---|---|---|
| never | 1 | لم أستخدمه أبداً |
| some of the days | 2 | بعض الأيام |
| most of the days | 3 | أغلب الأيام |

14.12 Do you have written instructions from your doctor on how to manage your asthma if it gets worse or if you have an attack?

14.12 هل لديك أية تعليمات كتابية من طبيبك عن كيفية معالجة أزمة الربو اذا تطورت، أو اذا تعرضت لأية نوبة؟

| | | |
|---|---|---|
| Yes | 1 | نعم |
| No | 2 | لا |

**FOR WOMEN ONLY - MEN GO TO Q15** **. للنساء فقط – الرجال انتقل إلى Q15**

Q 14.3 Have you ever noticed that your asthma got worse with your monthly cycle?
TICK ONE BOX ONLY

Q14.3هل لاحظتِ من قبل بأن الأزمة تطورت إلى الأسوأ خلال فترة الدورة الشهرية؟
ضع علامة على مربع واحد فقط

| | | |
|---|---|---|
| yes, in the week before my period | 1 | نعم خلال الأسبوع الذى يسبق الدورة |
| yes, during my period | 2 | نعم، خلال الدورة |
| yes, in the week after my period | 3 | نعم، فى الأسبوع الذى يلى الدورة |
| yes, another time of the month | 4 | نعم، فى وقتٍ آخر من الشهر |
| does not apply to me (i.e., amenorrhoeal) | 5 | لا ينطبق هذا علىّ (على سبيل المثال، تمر بفترة انقطاع الطمث) |
| No | 6 | لا |

14.14 Have you been pregnant (at least 25 weeks) since your asthma started?

14.14 هل كنتِ فى فترة الحمل (على الأقل 25 أسبوعاً) منذ أن بدأت الأزمة؟

| | | |
|---|---|---|
| Yes (Go to Q 14.14.1 ) | 1 | نعم (انتقل إلى Q 14.14.1) |
| No (Go to Q 15) | 2 | لا (انتقل إلىQ 15) |

14.14.1.What happened to your asthma during your pregnancies? TICK ONE BOX ONLY

14.14.1. كيف تطورت الأزمة خلال فترات الحمل؟ ضع علامة على مربع واحد فقط

| | | |
|---|---|---|
| got better | 1 | تحسنت |
| got worse | 2 | تطورت إلى الأسوأ |
| stayed the same | 3 | ظلت كما هى |
| not the same for all pregnancies | 4 | ليست كما هى خلال جميع فترات الحمل |
| don't know | 5 | لا أعرف |

15. Do you have any nasal allergies, including hay fever?

15. هل لديك أية حساسيات فى الأنف، بما فى ذلك حمى القش؟

| | | |
|---|---|---|
| Yes (Go to Q 15.1 ) | 1 | نعم (انتقل إلى Q 15.1) |
| No (Go to Q 16) | 2 | لا (انتقل إلىQ 16) |

15.1 How old were you when you first had hay fever or nasal allergy?

15.1 كم كان عمرك عندما أصابتك حمى القش أو أى حساسية فى الأنف؟

| | | |
|---|---|---|
| Age (years) | | العمر (أعوام) |

16. Have you ever had a problem with sneezing, or a runny or a blocked nose when you did not have a cold or the flu?

16. هل تعرضت من قبل لأية مشكلة تتعلق بالعطس، رشح أو انسداد بالأنف عندما لم تكن مصاباً بالبرد أو الإنفلونزا؟

| | | |
|---|---|---|
| Yes (Go to Q 16.1 ) | 1 | نعم (انتقل إلى Q 16.1) |
| No (Go to Q 17) | 2 | لا (انتقل إلى Q 17) |

16.1. Have you had a problem with sneezing or a runny or blocked nose when you did not have a cold or the flu in the last 12 months?

16.1 هل تعرضت من قبل لأية مشكلة تتعلق بالعطس، رشح أو انسداد بالأنف عندما لم تكن مصاباً بالبرد أو الإنفلونزا خلال الـ 12 شهراً الأخيرة؟

| | | |
|---|---|---|
| Yes (Go to Q 16.1.1 ) | 1 | نعم (انتقل إلى Q 16.1.1) |
| No (Go to Q 17) | 2 | لا (انتقل إلى Q 17) |

16.1.1. Has this nose problem been accompanied by itchy or watery eyes?

16.1.1 هل صاحب هذه المشكلة الخاصة بالأنف، شعوراً بالحكة أو بدموع فى العين؟

| | | |
|---|---|---|
| Yes | 1 | نعم |
| No | 2 | لا |

16.1.2. In which months of the year did this nose problem occur?

16.1.2 فى أى شهور من العام حدثت هذه المشكلة الخاصة بالأنف؟

| | | |
|---|---|---|
| Jan | 1 | يناير |
| Feb | 2 | فبراير |
| March | 3 | مارس |
| April | 4 | أبريل |
| May | 5 | مايو |
| June | 6 | يونيو |
| July | 7 | يوليو |
| August | 8 | أغسطس |
| Sep | 9 | سبتمبر |
| Oct | 10 | أكتوبر |
| Nov | 11 | نوفمبر |
| Dec | 12 | ديسمبر |

17. Have you used any medication to treat nasal disorders?

17. هل استخدمت أى أدوية لعلاج مشاكل الأنف؟

| | | |
|---|---|---|
| Yes (Go to Q 17.1 ) | 1 | نعم (انتقل إلى Q 17.1) |
| No (Go to Q 18) | 2 | لا (انتقل إلى Q 18) |

17.1 Show cards

17.1 اعرض البطاقات

Have you used any of the following nasal sprays for the treatment of your nasal disorder?

{SHOW LIST OF STEROID NASAL SPRAYS}: Nasonex/ Flixonase/ Rhoinocort

هل استخدمت أيٍ من بخاخات الأنف التالية لعلاج مشكلة الأنف الخاصة بك؟ [اعرض قائمة ببخاخات الأنف الستيرويدية]:نازونيكس / فليكسونيس / رينوكورت

| | | |
|---|---|---|
| Yes (Go to Q 17.1.1 ) | 1 | نعم (انتقل إلى Q 17.1.1) |
| No (Go to Q 17.2) | 2 | لا (انتقل إلى Q 17.2) |

17.1.1 How many years have you been taking this sort of nasal spray?

17.1.1 منذ متى وأنت تستخدم هذا النوع من بخاخات الأنف؟

| | | |
|---|---|---|
| Number of years | | عدد الأعوام |

17.1.2 Have you used any of these nasal sprays in the last 12 months?

17.1.2 هل استخدمت أيٍ من بخاخات الأنف هذه خلال الـ 12 شهراً الماضية؟

| | | |
|---|---|---|
| Yes | 1 | نعم |
| No | 2 | لا |

17.2 Show card

Have you used any of the following pills, capsules, or tablets for the treatment of your nasal disorder?

{SHOW LIST OF ANTIHISTAMINES}: Aerius/ Claritin/ Xyzal/ Telfast/ Polaramine/ Piriton

17.2 اعرض البطاقات

هل استخدمت أيٍ من الحبات، الكبسولات، أو الأقراص التالية لعلاج المشكلة الخاصة بأنفك؟ [اعرض قائمة بمضادات الهيستامين التالية]: ايريوس/ كلاريتين/ اكسيزال/ تلفاست/ بولارامين/ بيريتون

| | | |
|---|---|---|
| Yes (Go to Q 17.2.1 ) | 1 | نعم (انتقل إلى Q 17.2.1) |
| No (Go to Q 18) | 2 | لا (انتقل إلى Q 18) |

17.2.1 How many years have you been taking these sort of pills, capsules or tablets?

17.2.1 منذ متى وأنت تتناول هذه الأنواع من الحبات، الكبسولات أو الأقراص؟

| | | |
|---|---|---|
| Number of years | | عدد الأعوام |

17.2.2 Have you used any of these pills, capsules or tablets in the last 12 months?

17.2.2 هل استخدمت أيٍ من هذه الحبات، الكبسولات أو الأقراص خلال الـ 12 شهراً الأخيرة؟

| | | |
|---|---|---|
| Yes | 1 | نعم |
| No | 2 | لا |

18. Have you ever had eczema or any kind of skin allergy?

18. هل سبق لك وأن تعرضت لأى مرض جلدى أو لأى نوع من الحساسيات فى الجلد؟

| | | |
|---|---|---|
| Yes | 1 | نعم |
| No | 2 | لا |

19. Have you ever had an itchy rash that was

19. هل سبق وأن أصابك أى طفح جلدى يسبب الحكة، كان يأتى

coming and going for at least 6 months? ويذهب لمدة 6 أشهر على الأقل؟

| | | |
|---|---|---|
| Yes (Go to Q 19.1 ) | 1 | نعم (انتقل إلى Q 19.1) |
| No (Go to Q 20 ) | 2 | لا (انتقل إلى Q 20) |

19.1. Have you had this itchy rash in the last 12 months?

19.1 هل أصابك هذا الطفح الجلدى الذى يسبب الحكة خلال الـ 12 شهراً الماضية؟

| | | |
|---|---|---|
| Yes (Go to Q 19.1.1 ) | 1 | نعم (انتقل إلى Q 19.1.1) |
| No (Go to Q 20 ) | 2 | لا (انتقل إلى Q 20) |

19.1.1. Has this itchy rash at any time affected any of the following places: the folds of the elbows, behind the knees, in front of the ankles under the buttocks or around the neck, ears or eyes

19.1.1 هل آثر هذا الطفح الجلدى الذى يسبب الحكة فى أى وقت على أيٍ من الأجزاء التالية: ثنيات المرفق، خلف الركبتين، أمام الكاحل، تحت الأرداف أو حول العنق، الأذن أو العينين

| | | |
|---|---|---|
| Yes | 1 | نعم |
| No | 2 | لا |

20. Have you ever had any difficulty with your breathing after taking medicines?

20. هل سبق وأن شعرت بأية صعوبة فى التنفس بعد تناولك للأدوية؟

| | | |
|---|---|---|
| Yes (Go to Q 20.1.2 ) | 1 | نعم (انتقل إلى Q 20.1.2) |
| No (Go to Q 21 ) | 2 | لا (انتقل إلى Q 21) |

20.1-2 Show card
Which medicines?

20.1-2 اعرض البطاقات

أية أدوية؟

| | | |
|---|---|---|
| Aspirin | 1 | أسبرين |
| Beta-blockers | 2 | مثبطات البيتا |
| Non-steroidal anti-inflammatory agents | 3 | الأدوية غير الستيرويدية المضادة للالتهابات |
| Mixture of the above | 4 | مزيج من الأدوية المذكورة بالأعلى |
| Not known | 5 | غير معروف |

21. How old was your mother when you were born?

21. كم كان عمر والدتك عند ولادتك؟

| | | |
|---|---|---|
| Age (years) | | العمر (أعوام) |

22. How many times did you move house during the first five years of your life?

22. كم مرة انتقلت فيها من منزلك خلال أول خمسة أعوام من حياتك؟

| | | |
|---|---|---|
| Number of times | | عدد المرات |

23. Were you hospitalised before the age of two years for lung disease?

23. هل دخلت المستشفى للعلاج من مرض بالرئة قبل أن تبلغ عامين؟

| | | |
|---|---|---|
| Yes | 1 | نعم |
| No | 2 | لا |

24. At what age did you first attend a school, play school, day care or nursery?

24. فى أى عمر بدأت فى الذهاب للمدرسة، أو لمدرسة تعمل حتى نصف اليوم، للحضانة، أو لحضانة خاصة بالأطفال الرضع؟

| | | |
|---|---|---|
| Age (years) | | العمر (أعوام) |

25. How many other children regularly slept in your bedroom before you were five years old?

25. كم عدد الأطفال الآخرين الذين كانوا عادةً ما ينامون فى غرفة نومك قبل أن تبلغ خمسة أعوام؟

| | | |
|---|---|---|
| Number of other children | | عدد الأطفال الآخرين |

**I would now like to ask you some questions on the type of jobs that you have done. I am interested in each one of the jobs that you have done for more than 3 consecutive months since the time we last contacted you (in 1991/2). These jobs may be outside the house or at home, full time or part time, paid or not paid, including self employment, for example in a family business. Please include part time jobs only if you had been doing them for more than 8 hours per week.**

**أود الآن أن أسألك بعض الأسئلة عن نوعية الوظائف التى كنت تشغلها. فأنا مهتم بكل وظيفة من الوظائف التى كنت تشغلها لأكثر من 3 أشهر متتالية منذ آخر مرة قمنا فيها بالاتصال بك (فى 1991/ 2).**
**يمكن أن تكون هذه الوظائف خارج المنزل أو بالمنزل، دوام كامل أو دوام جزئى، وفى مقابل أجر أو بدون. وتشمل العمل لحساب نفسك، على سبيل المثال عمل عائلى. من فضلك قُم بإدارج الوظائف بنظام الدوام الجزئى، فقط اذا كنت تعمل فيها لأكثر من 8 ساعات فى الأسبوع.**

26. Are you currently ( TICK ONE BOX ONLY )

26. هل أنت حالياً (ضع علامة على مربع واحد فقط)

| | | |
|---|---|---|
| Employed (including military service) | 1 | تعمل (بما فى ذلك الخدمة العسكرية) |
| Self employed | 2 | تعمل لحساب نفسك |
| Unemployed, looking for work | 3 | لا تعمل، تبحث عن عمل |
| Not working because of poor health | 4 | لا تعمل لسبب صحى |
| Full-time house-person | 5 | ربة منزل |
| Full time student | 6 | طالب بدوام كلى |
| Retired | 7 | متقاعد |
| Other | 8 | أخرى |

| | |
|---|---|
| IF EMPLOYED OR SELF EMPLOYED OR A FULL TIME HOURSEPERSON GO TO Q28 ELSE GO TO Q27 | انتقل إلى Q28 اذا كان يعمل لحساب نفسه أو يعمل بدوام كامل أو ربة منزل<br>أخرى انتقل إلى Q27 |

27. Have you been employed in any job for three continuous months or longer?

27. هل كنت تعمل فى أية وظيفة لمدة 3 أشهر متواصلة أو أكثر؟

| | | |
|---|---|---|
| Yes (Go to Q 28 ) | 1 | نعم (انتقل إلى Q 28) |
| No (Go to Q 29 ) | 2 | لا (انتقل إلى Q 29) |

| 28. If you had more than one job in the same company, or if you were doing more than one job at the same time, we would like to talk about them separately. Please start with your current or last job. | 28. اذا كنت تشغل أكثر من وظيفة واحدة فى نفس الشركة، أو اذا كنت تعمل فى أكثر من وظيفة فى نفس الوقت، نود أن نتحدث عن كلٍ منها بشكلٍ منفصل. من فضلك ابدأ بوظيفتك الحالية أو الأخيرة. |
|---|---|

| | |
|---|---|
| Q 28.1.<br>What is (was) the title of your current (last) job? | 28.1 ما (ماذا كان) المسمى الوظيفى لوظيفتك الحالية (الأخيرة)؟ |
| Q 28.2.<br><br>What did the firm, company or rganization do or what services did it provide? | Q28.2<br><br>ما المجال الذى تعمل فيه هذه الشركة أو المؤسسة أو ما الخدمات التى تقدمها؟ |
| Q 28.3.<br><br>In what month and year did you start working in this job? | Q 28.3<br><br>فى أى شهر وعام بدأت العمل فى هذه الوظيفة؟ |
| Q28.4.<br><br>In what month and year did you stop working in this job? | Q28.4<br><br>فى أى شهر وعام توقفت عن العمل فى هذه الوظيفة؟ |

| | **28.1** | **28.2** | **28.3** | | **28.4** | | |
|---|---|---|---|---|---|---|---|
| JOB | Occupation /Profession<br>المهنة/ الوظيفة | Industry<br>المجال | Month<br>شهر | Year<br>عام | Month<br>شهر | Year<br>عام | الوظيفة |
| JOB 1 | | | | | | | وظيفة 1 |

| | | | | | |
|---|---|---|---|---|---|
| JOB 2 | | | | | وظيفة 2 |
| JOB 3 | | | | | وظيفة 3 |
| JOB 4 | | | | | وظيفة 4 |
| JOB5 | | | | | وظيفة 5 |
| JOB 6 | | | | | وظيفة 6 |

29. Have any of these jobs ever made your chest tight or wheezy?

29. هل سبق وأن سببت لك أية وظيفة من هذه الوظائف شعوراً بضيق أو أزيز في صدرك؟

| | | |
|---|---|---|
| Yes (tick no or yes for each job)) | 1 | نعم(اختر لا أو نعم لكل وظيفة) |
| No (Go to Q 31 ) | 2 | لا (انتقل إلىQ 31) |

| | |
|---|---|
| IF YES, (tick no or yes for each job) | اذا كانت الإجابة نعم، (اختر لا أو نعم لكل وظيفة) |

| | *Yes* *نعم* | *No* *لا* | |
|---|---|---|---|
| JOB1 | *1* | *2* | وظيفة 1 |
| JOB2 | *1* | *2* | وظيفة 2 |
| JOB3 | *1* | *2* | وظيفة 3 |
| JOB4 | *1* | *2* | وظيفة 4 |
| JOB5 | *1* | *2* | وظيفة 5 |
| JOB6 | *1* | *2* | وظيفة 6 |

30. Have you had to leave any of these jobs because they affected your breathing?

30. هل اضطررت لأن تترك أيٍ من هذه الوظائف لأنها آثرت على عمليةٍ تنفسك؟

| | | |
|---|---|---|
| Yes (tick no or yes for each job)) | 1 | نعم(اختر لا أو نعم لكل وظيفة) |
| No (Go to Q31) | 2 | لا (انتقل إلىQ 31) |

| | |
|---|---|
| IF YES, (tick no or yes for each job) | اذا كانت الإجابة نعم، (اختر لا أو نعم لكل وظيفة) |

| | *Yes* *نعم* | *No* *لا* | |
|---|---|---|---|
| JOB1 | *1* | *2* | وظيفة 1 |
| JOB2 | *1* | *2* | وظيفة 2 |

| | | | |
|---|---|---|---|
| JOB3 | *1* | *2* | وظيفة 3 |
| JOB4 | *1* | *2* | وظيفة 4 |
| JOB5 | *1* | *2* | وظيفة 5 |
| JOB6 | *1* | *2* | وظيفة 6 |

31.Have you been involved in an accident at home, work or elsewhere that exposed you to high levels of vapours, gas, dust or fumes?

31. هل تعرضت لأى حادث سواء بالمنزل، بالعمل أو بأى مكانٍ آخر حيث استنشقت كميات كبيرة من البخار أو الغاز أو الغبار\ التراب أو الدخان؟

| | | |
|---|---|---|
| Yes (goto Q31.1) | 1 | نعم (انتقل إلىQ 31.1) |
| No (go to Q32) | 2 | لا (انتقل إلىQ 32) |

31.1 Did you experience respiratory symptoms immediately following this exposure?

31.1 هل صادفتك أى أعراض تنفسية بعد تعرضك لهذا الحادث؟

| | | |
|---|---|---|
| Yes (goto Q31.1.1) | 1 | نعم (انتقل إلىQ 31.1.1) |
| No (go to Q32) | 2 | لا (انتقل إلىQ 32) |

31.1.1 Could you describe to me what it was?

31.1.1 هل يمكنك أن تصف لى ماذا كانت هذه الأعراض؟

| | |
|---|---|
| Description of respiratory symptoms following exposure | وصف الأعراض التنفسية بعد التعرُض للحادث: |

Centres performing the extra occupational modules should at this point introduce the modular introductory questionnaire and complete modules as appropriate.

عند هذه النقطة، يجب أن تقدم المراكز التى تقوم بالأقسام المهنية الإضافية، الاستمارة التمهيدية. وعليها أن تقوم أيضاً باستكمال الأقسام وفقاً لما يكون مناسباً

32. At what age did you complete full time education?

32. فى أى عمر أكملت التعليم الأساسى؟

| | | |
|---|---|---|
| Age (years) | | العمر (أعوام) |

| | |
|---|---|
| If full time student enter 88 | ادخِل 88، اذا كان طالباً بنظام الدوام الكلى |

33. How often do you usually exercise so much that you get out of breath
or sweat? TICK ONE BOX ONLY

33. ما المعدل الذى دائماً ما تقوم من خلاله باختبار تنفسك أو باختبار كمية العرق الذى تفرزه؟ ضع علامة على مربع واحد فقط

| | | |
|---|---|---|
| every day | 1 | كل يوم |
| 4-6 times a week | 2 | 6-4 مرات فى الأسبوع |

| | | |
|---|---|---|
| 2-3 times a week | 3 | 2-3 مرات فى الأسبوع |
| once a week | 4 | مرة فى الأسبوع |
| once a month | 5 | مرة فى الشهر |
| less than once a month | 6 | أقل من مرة فى الشهر |
| never | 7 | أبداً |

34. How many hours a week do you usually exercise so much that you get out of breath or sweat? TICK ONE BOX ONLY

34. كم ساعة فى الأسبوع تقوم خلالها باختبار تنفسك أو باختبار كمية العرق الذى تفرزه؟ ضع علامة على مربع واحد فقط

| | | |
|---|---|---|
| none | 1 | لا يوجد |
| about ½ hr | 2 | حوالى ½ ساعة |
| about 1 hour | 3 | حوالى ساعة |
| about 2-3 hours | 4 | من 2-3 ساعات تقريباً |
| about 4-6 hours | 5 | من 4-6 ساعات تقريباً |
| 7 hours or more | 6 | 7 ساعات أو أكثر |

35. Do you avoid taking vigorous exercise because of wheezing or asthma?

35. هل تتجنب القيام باختبار قوى أو عنيف خوفاً من أن تتعرض لأزمة أو لأزيز فى صدرك؟

| | | |
|---|---|---|
| Yes | 1 | نعم |
| No | 2 | لا |

36. When was your present home built?

36. متى تم بناء المنزل الحالى الذى تعيش فيه؟

| | | |
|---|---|---|
| Year | | عام |

37.1. How many years have you lived in your current home?

37.1. منذ متى وأنت تعيش فى منزلك الحالى؟

| | | |
|---|---|---|
| Number of years | | عدد الأعوام |

37.2 Which best describes the building in which you live? TICK ONE BOX ONLY

ما أفضل شيء يصف المنزل الذى تعيش فيه؟ ضع علامة على مربع واحد فقط

| | | |
|---|---|---|
| a) a mobile home or trailer? | 1 | a) منزل متحرك أو عربة مقطورة؟ |
| b) a one family house detached from any other house? | 2 | b) منزل مخصص لعائلة واحدة منفصل عن أى منزل آخر؟ |
| c) a one family house attached to one or more houses? | 3 | c) منزل مخصص لعائلة واحدة متصل بمنزل واحد أو بمنازل |

| | | |
|---|---|---|
| | | متعددة؟ |
| d) a building for two families? | 4 | d) مبنى مخصص لعائلتين؟ |
| e) a building for three or four families? | 5 | e) مبنى مخصص لثلاث أو أربع عائلات؟ |
| f) a building for five or more families? | 6 | f) مبنى مخصص لخمس عائلات أو أكثر؟ |
| g) a boat, tent or van | 7 | g) مركب، خيمة أو عربة للنقل |
| e) other: ___________________________ | 8 | e) أخرى: _________________ |

38. What kind of air cooling do you have at home? 38. ما نوع تكييف الهواء لديك فى المنزل؟

| | | *Yes* *نعم* | *No* *لا* | |
|---|---|---|---|---|
| Fan | 1 | *1* | *2* | مروحة |
| Air purifiers/ dehumidifier | 2 | *1* | *2* | منقى للهواء/ مزيل للرطوبة |
| Window air conditioner | 3 | *1* | *2* | تكييف شباك |
| Central air conditioner | 4 | *1* | *2* | تكييف مركزى |
| Central Heating | 5 | *1* | *2* | مدفأة مركزية |
| Windows Only | 6 | *1* | *2* | شباك فقط |

39. Which of the following appliances do you use for heating or for hot water? 39. أيٍ من الأجهزة التالية تستخدم للتسخين أو للحصول على مياه ساخنة؟

| | | Yes نعم | No لا | | |
|---|---|---|---|---|---|
| open coal, coke or wood fire | 1 | *1* | *2* | 1 | إشعال النار عبر استخدام الفحم الطبيعى، فحم الكوك أو الخشب |
| open gas fire | 2 | *1* | *2* | 2 | إشعال النار من خلال الغاز |
| electric heater | 3 | *1* | *2* | 3 | سخان كهربائى |
| paraffin heater | 4 | *1* | *2* | 4 | سخان كيروسين |
| gas-fired boiler | 5 | *1* | *2* | 5 | غلاية تعمل بالغاز |
| oil-fired boiler | 6 | *1* | *2* | 6 | غلاية تعمل بالزيت |
| portable gas heater | 7 | *1* | *2* | 7 | سخان غاز متنقل |
| other: ___________________________ | 8 | *1* | *2* | 8 | أخرى: ___________________________ |

| | | | | | |
|---|---|---|---|---|---|
| ___________ | | | | | ________ |

40. What kind of stove do you mostly use for cooking? TICK ONE BOX ONLY

40. ما نوع الموقد الذى غالباً ما تستخدمه للطهى؟ ضع علامة على مربع واحد فقط

| | | |
|---|---|---|
| a) coal, coke or wood (solid fuel)? | 1 | a) فحم، فحم الكوك أو الخشب (وقود صلب)؟ |
| b) gas (gas from the mains)? | 2 | b) غاز (غاز من المنبع)؟ |
| c) electric? | 3 | c) كهربائى؟ |
| d) paraffin (kerosene)? | 4 | d) بارافين (كيروسين)؟ |
| e) microwave | 5 | e) ميكروويف |
| f) gas (gas from bottles or other non-mains source) | 6 | f) غاز (من خلال الأنابيب، أو من أى مصدر آخر ليس من المنبع) |
| g) other: ______________________________ ___________ | 7 | g) أخرى:____________________ |

Q40.1 IF YOU USE GAS FOR COOKING Which of the following do you have?

Q 40.1 اذا كنت تستخدم الغاز فى الطهى، أيٍ من الأدوات التالية لديك؟

| | Yes<br>نعم | No<br>لا | |
|---|---|---|---|
| gas hob | 1 | 2 | عين الموقد |
| gas oven | 1 | 2 | فرن الموقد |

Q41. What kind of stove was mostly used for cooking in the home you lived in when you were five years old? TICK ONE BOX ONLY

Q41. ما نوع الموقد الذى كان غالباً ما يُستخدم للطهى، فى المنزل الذى كنت تعيش فيه عندما كان عمرك خمسة أعوام؟ ضع علامة على مربع واحد فقط

| | | |
|---|---|---|
| a) coal, coke or wood (solid fuel)? | 1 | a) فحم، فحم الكوك أو الخشب (وقود صلب)؟ |
| b) gas (gas from the mains)? | 2 | b) غاز (غاز من المنبع)؟ |
| c) electric? | 3 | c) كهربائى؟ |
| d) paraffin (kerosene)? | 4 | d) بارافين (كيروسين)؟ |
| e) microwave | 5 | e) ميكروويف |
| f) gas (gas from bottles or other non-mains source) | 6 | f) غاز (من خلال الأنابيب، أو من أى مصدر آخر ليس من المنبع) |
| g) other: | 7 | g) أخرى:____________________ |

| ____________________________ | | |
|---|---|---|

Q42. On average how long have you spent cooking with your stove each day over the last four weeks?

Q.42 فى المتوسط، كم أمضيت من وقت للطهى باستخدام الموقد فى كل يوم خلال الأربعة أسابيع الماضية؟

| | | |
|---|---|---|
| Average time spent cooking (minutes) | | متوسط الوقت الذى أمضاه فى الطهى (دقائق) |

Q43. Over the last four weeks when you were cooking did you have a kitchen door or window to the outside air open TICK ONE BOX ONLY

Q43. هل كان لديك باب بالمطبخ أو نافذة، مفتوحان لدخول الهواء الخارجى ضع علامة على مربع واحد فقط

| | | |
|---|---|---|
| a) most of the time | 1 | a) فى معظم الوقت |
| b) some of time | 2 | b) بعض الوقت |
| c) rarely (or only occasionally) | 3 | c) نادراً (أو فقط قليلاً) |
| d) I do not have a door or window that opens to the outside in my kitchen | 4 | d) ليس لدىّ باب أو نافذة بالمطبخ مفتوحان على الخارج |

Q44. Do you have an extractor fan (or chimney) over the cooker?

Q44. هل لديك مروحة لاستخراج الهواء (أو مدخنة) أعلى الموقد؟

| | | |
|---|---|---|
| Yes ( Go to 44.1) | 1 | نعم (انتقل إلى 44.1) |
| No ( Go To Q 45) | 2 | لا (انتقل إلى Q 45) |
| DK ( Go To Q 45) | 3 | لا أعرف (انتقل إلى Q 45) |

Q44.1 When cooking, do you use the fan TICK ONE BOX ONLY

Q 44.1 هل تستخدم هذه المروحة عند قيامك بالطهى، ضع علامة على مربع واحد فقط

| | | |
|---|---|---|
| a) all of the time? | 1 | a) طوال الوقت؟ |
| b) some of the time? | 2 | c) بعض الوقت؟ |
| c) none of the time? | 3 | c) لا أستخدمها فى أى وقت؟ |

Q44.2 Does the fan take the fumes outside the house?

Q 44.2 هل تقوم هذه المروحة بإخراج الدخان خارج المنزل؟

| | | |
|---|---|---|
| Yes | 1 | نعم |
| No | 2 | لا |
| DK | 3 | لا أعرف |

Q45. Does the room which you use most at home during the day (MA)

Q.45 هل أكثر غرفة تكون بها طوال اليوم بالمنزل (إجابات متعددة)

| | Yes<br>نعم | No<br>لا | |
|---|---|---|---|
| Q45.1<br>have fitted carpets covering the whole floor? | 1 | 2 | Q45.1<br>تحتوى على سجاد يغطى الأرضية بأكملها؟ |
| Q45.2<br>contain rugs? | 1 | 2 | Q 45.2<br>تحتوى على سجاد صغير (مشايات)؟ |
| Q45.3<br>have double glazing? | 1 | 2 | Q45.3<br>تحتوى على زجاج مزدوج؟ |

Q46. How old is the oldest carpet or rug in the room which you use most at home during the day? TICK ONE BOX ONLY

Q.46 كم يبلغ عمر أقدم سجادة أو مشاية فى أكثر غرفة تكون بها طوال اليوم بالمنزل ضع علامة على مربع واحد فقط

| | | |
|---|---|---|
| a) less than one year | 1 | a) أقل من عام |
| b) 1-5 years old | 2 | b) 1-5 أعوام |
| c) more than 5 years old | 3 | c) أكثر من 5 أعوام |

Q47. On what floor is the room which you use most at home during the day?(The lowest floor of a building is 00)

Q.47 فى أى طابق تقع أكثر غرفة تكون بها طوال اليوم بالمنزل (أول طابق بالمبنى سيكون 00)

| | | |
|---|---|---|
| Floor | | الطابق |

Q48. Does your bedroom

Q.48 هل غرفة نومك

| | Yes<br>نعم | No<br>لا | |
|---|---|---|---|
| **Q48.1**<br>have fitted carpets covering the whole floor? | 1 | 2 | **Q48.1**<br>تحتوى على سجاد يغطى الأرضية بأكملها؟ |
| **Q48.2**<br>contain rugs? | 1 | 2 | **Q 48.2**<br>تحتوى على سجاد صغير (مشايات)؟ |
| **Q48.3**<br>have double glazing? | 1 | 2 | **Q 48.3**<br>تحتوى على زجاج مزدوج؟ |

Q49. How old is the oldest carpet or rug in your bedroom TICK ONE BOX ONLY

Q.49 كم يبلغ عمر أقدم سجادة أو مشاية فى غرفة نومك اختر مربع واحد فقط

| | | |
|---|---|---|
| a) less than one year | 1 | a) أقل من عام |
| b) 1-5 years old | 2 | b) 1-5 أعوام |
| c) more than 5 years old | 3 | c) أكثر من 5 أعوام |

Q50. How old is your mattress TICK ONE BOX ONLY

Q50 كم يبلغ عمر المرتبة الخاصة بك؟ اختر مربع واحد فقط

| | | |
|---|---|---|
| a) less than one year | 1 | a) أقل من عام |
| b) 1-5 years old | 2 | b) 1-5 أعوام |
| c) more than 5 years old | 3 | c) أكثر من 5 أعوام |

Q51. What floor of the building is your edroom on? (lowest=00)

Q.51 فى أى طابق تقع غرفة نومك؟(أول طابق بالمبنى سيكون 00)

| | | |
|---|---|---|
| Floor | | الطابق |

Q52. Do you sleep with the windows open at night during winter?

Q52. هل تنام والنوافذ مفتوحة أثناء الليل فى الشتاء؟

| | | |
|---|---|---|
| Yes ( Go to 52.1) | 1 | نعم (انتقل إلى 52.1) |
| No ( Go To Q 53) | 2 | لا (انتقل إلى Q 53) |

Q52.1 Do you sleep with the windows open TICK ONE BOX ONLY

Q 52.1 هل تنام والنوافذ مفتوحة ضع علامة على مربع واحد فقط

| | | |
|---|---|---|
| a) all of the time? | 1 | a) طوال الوقت؟ |
| b) sometimes? | 2 | b) بعض الوقت؟ |
| c) only occasionally? | 3 | c) قليلاً فقط؟ |

Q53. Has there been any water damage to the building or its contents, for example, from broken pipes, leaks or floods?

Q.53 هل وقع أى ضرر للمبنى بفعل المياه أو محتوياتها، مثلاً بفعل أنابيب مكسورة، تسريب أو فيضان؟

| | | |
|---|---|---|
| Yes ( Go to 53.1) | 1 | نعم (انتقل إلى 53.1) |
| No ( Go to 54 ) | 2 | لا ( انتقل إلى 54) |
| DK ( Go to 54 ) | 3 | لا أعرف ( انتقل إلى 54) |

Q53.1 Has there been any water damage in the last 12 months

Q 53.1 هل وقع أى ضرر بفعل المياه خلال الـ 12 شهراً الماضية؟

| | | |
|---|---|---|
| Yes | 1 | نعم |
| No | 2 | لا |
| DK | 3 | لا أعرف |

Q54. Within the last 12 months have you had wet or damp spots on surfaces inside your home other than in the basement (for example on walls, wall paper, ceilings or carpets)?

Q .54 هل كان لديك بقع مبللة أو ناتجة عن رطوبة على الأسطح داخل منزلك غير تلك الموجودة بالقبو، خلال الـ 12 شهراً الأخيرة (على سبيل المثال على الحوائط، ورق الحائط، الأسقف أو السجاد)؟

| | | |
|---|---|---|
| Yes | 1 | نعم |
| No | 2 | لا |

Q55. Has there ever been any mould or mildew on any surface, other than food, inside the home?

Q .55 هل كان هناك أى فطريات أو عفن على أى أسطَح داخل المنزل، غير تلك الناتجة عن الطعام؟

| | | |
|---|---|---|
| Yes | 1 | نعم |
| No | 2 | لا |
| DK | 3 | لا أعرف |

Q55.1.1-6 Which rooms have been affected?

Q 55.1.1-6 ما الغرف التى تأثرت؟

| | Yes<br>نعم | No<br>لا | |
|---|---|---|---|
| **Q55.1.1**<br>bathroom(s) | 1 | 2 | **Q 55.1.1**<br>الحمام (الحمامات) |
| **Q55.1.2**<br>bedroom(s) | 1 | 2 | **Q 55.1.2**<br>غرفة (غرف) النوم |
| **Q55.1.3**<br>living area(s) | 1 | 2 | Q 55.1.3 غرفة (غرف) المعيشة |
| **Q55.1.4**<br>kitchen | 1 | 2 | **Q 55.1.4**<br>المطبخ |
| **Q55.1.5** | 1 | 2 | **Q 55.1.5** |

| | | | |
|---|---|---|---|
| basement or attic | | | القبو أو العلية |
| **Q55.1.6**<br><br>other: ____________________<br>___________ | | | 55.1.6 أخرى:<br>_______________ |

Q55.2 Has there been mould or mildew (fungus) on any surfaces inside the home in the last 12 months?

Q 55.2 هل كان هناك أى فطريات أو عفن على أى أسطَح داخل المنزل، خلال الـ 12 شهراً الأخيرة؟

| | | |
|---|---|---|
| Yes | 1 | نعم |
| No | 2 | لا |
| | | |

'This scale looks like a thermometer; it allows you to rate your personal opinion regarding the following question on annoyance from air pollution. You can indicate your level of annoyance on this scale between 0 and 10 where 0 mean does not annoy at all' and 10 means intolerable annoyance.'

"يشبه المقياس التالى جهاز قياس درجة الحرارة: ويتيح لك أن تقوم بتقييم رأيك الشخصى فيما يتعلق بالسؤال التالى عن مدى انزعاجك من تلوث الهواء. يمكنك أن توضح مدى انزعاجك من خلال استخدام هذا المقياس من 0 إلى 10، حيث يفيد اختيار 0 بأنه "لا يسبب إزعاجاً على الإطلاق" بينما يفيد اختيار 10 بأنه "يسبب إزعاجاً بشكلٍ غير محتمل".

Q56. How much are you annoyed by outdoor air pollution (from traffic, industry, etc.) if you keep the windows open?

Q .56 إلى أى مدى يضايقك تلوث الهواء الخارجى (الناتج عن الزحام، المناطق الصناعية، إلخ) اذا لم تقم بإغلاق النوافذ؟

| | | | | | | | | | | |
|---|---|---|---|---|---|---|---|---|---|---|
| Intolerable Annoyance يسبب إزعاجاً بشكلٍ غير محتمل | ←→ | | | | | | | | | Does not Annoy at all لا يسبب إزعاجاً على الإطلاق |
| 10 | 9 | 8 | 7 | 6 | 5 | 4 | 3 | 2 | 1 | 0 |

Q57.1 How much were you annoyed by outdoor air pollution (from traffic, industry, etc.) in your previous home, if you kept the windows open?

Q57.1 إلى أى مدى كان يضايقك تلوث الهواء الخارجى الناتج عن الزحام، المناطق الصناعية، إلخ) فى منزلك السابق، اذا لم تقم بإغلاق النوافذ؟

| | | |
|---|---|---|
| Intolerable Annoyance يسبب إزعاجاً بشكلٍ غير محتمل | ←→ | Does not Annoy at all لا يسبب إزعاجاً على |

| | | | | | | | | | | الإطلاق |
|---|---|---|---|---|---|---|---|---|---|---|
| 10 | 9 | 8 | 7 | 6 | 5 | 4 | 3 | 2 | 1 | 0 |

Q57.2 Is there a construction/industrial site near your:

Q 57.2 هل هناك أى موقع تحت الإنشاء/ منطقة صناعية بالقرب من:

| | Yes<br>نعم | No<br>لا | |
|---|---|---|---|
| 57.2.1 Home | 1 | 2 | 57.2.1 المنزل |
| 57.2.2 Work | 1 | 2 | 57.2.2 العمل |
| 57.2.3 School | 1 | 2 | 57.2.2 المدرسة |

Q58. How often do cars pass your house? TICK ONE BOX ONLY

Q.58 ما معدل مرور السيارات بالقرب من منزلك؟ ضع علامة على مربع واحد فقط

| | | |
|---|---|---|
| a) constantly | 1 | a) دائماً |
| b) frequently | 2 | b) كثيراً |
| c) seldom | 3 | c) نادراً |
| d) never | 4 | d) أبداً |

Q59. How often do heavy vehicles (e.g. trucks/buses) pass your house? TICK ONE BOX ONLY

Q.59 ما معدل مرور السيارات الثقيلة (مثل الشاحنات/ الحافلات) بالقرب من منزلك؟ ضع علامة على مربع واحد فقط

| | | |
|---|---|---|
| a) constantly | 1 | a) دائماً |
| b) frequently | 2 | b) كثيراً |
| c) seldom | 3 | c) نادراً |
| d) never | 4 | d) أبداً |

Q60. Have you taken any of the following measures to reduce allergen or exposure to allergen in your home?

Q .60 هل اتبعت أيٍ من الإجراءات التالية حتى تقلل من إمكانية تعرضك لأى مادة تثير الحساسية فى منزلك؟

| | Yes<br>نعم | No<br>لا | |
|---|---|---|---|
| **Q60.1**<br>changed from carpet to a wooden or other smooth surface on floor of the room you use most | 1 | 2 | **Q 60.1**<br>قُمت بتغيير السجاد الموجود فى أرضية أكثر غرفة تمكث بها، واستبدلته بأرضيات خشبية أو بأى سطح آخر يتسم بالنعومة |
| **Q60.2**<br>changed from carpet to a wooden or to a smooth surface on floor of your bedroom | 1 | 2 | **Q 1 60.2**<br>قُمت بتغيير السجاد الموجود فى أرضية غرفة نومك، واستبدلته بأرضيات خشبية أو بأى سطح آخر يتسم بالنعومة |
| Q60.3<br>bought a new carpet for the room you use most | 1 | 2 | **Q 60.3**<br>قمت بشراء سجادة جديدة لأكثر غرفة تمكث بها |
| **Q60.4**<br>bought a new carpet for your bedroom | 1 | 2 | Q **60.4**<br>قمت بشراء سجادة جديدة لغرفة نومك |
| **Q60.5**<br>used antidust-mite sprays | 1 | 2 | **Q 60.5**<br>استخدمت بخاخات مضادة للغبار والعث |
| **Q60.6**<br>put an allergy-proof cover on your mattress | 1 | 2 | **Q 60.6**<br>وضعت غطاء مضاد للحساسية على المرتبة الخاصة بك |
| **Q60.7**<br>sold, given away or destroyed a pet dog or cat | 1 | 2 | **Q 60.7**<br>قُمت ببيع، بإبعاد أو بالقضاء على كلب منزلى أو قطة منزلية |

Q61. Do you keep a cat? Q.61 هل لديك قطة؟

| | | |
|---|---|---|
| Yes ( Go to 61.1) | 1 | نعم (انتقل إلى 61.1) |
| No ( Go to 62 ) | 2 | لا ( انتقل إلى 62) |

| | Yes<br>نعم | No<br>لا | |
|---|---|---|---|
| **Q61.1**<br>Is your cat (are your cats) allowed inside the house? | 1 | 2 | **Q61.1**<br>هل تسمح لقطتك (لقططك) بالدخول إلى المنزل؟ |
| **Q61.2**<br>Is your cat (are your cats) allowed in the bedroom? | 1 | 2 | **Q61.2**<br>هل تسمح لقطتك (لقططك) بالدخول إلى غرفة النوم؟ |

Q62. Do you keep a dog? ‏Q .62 هل لديك كلب؟

| | | |
|---|---|---|
| Yes ( Go to 62.1) | 1 | نعم (انتقل إلى 62.1) |
| No ( Go to 63 ) | 2 | لا ( انتقل إلى 63) |

| | Yes<br>نعم | No<br>لا | |
|---|---|---|---|
| **Q62.1**<br>Is your dog (are your dogs) allowed inside the house? | 1 | 2 | **Q62.1**<br>هل تسمح لكلبك (كلابك) بالدخول إلى المنزل؟ |
| **Q62.2**<br>Is your dog (are your dogs) allowed in your bedroom? | 1 | 2 | **Q62.2**<br>هل تسمح لكلبك (كلابك) بالدخول إلى غرفة النوم؟ |

63. Do you keep any birds? ‏.63 هل تحتفظ بأى طيور؟

| | | |
|---|---|---|
| Yes ( Go to 63.1) | 1 | نعم (انتقل إلى 63.1) |
| No ( Go to 64 ) | 2 | لا ( انتقل إلى 64) |

Q63.1 Are any of these birds kept inside the house? ‏Q 63.1 هل تحتفظ بأيٍ من هذه الطيور داخل المنزل؟

| | | |
|---|---|---|
| Yes | 1 | نعم |
| No | 2 | لا |

Q64. Was there a cat in your home? ‏Q .64 هل كانت لديك قطة فى منزلك؟

| | Yes<br>نعم | No<br>لا | DK<br>لا أعرف | |
|---|---|---|---|---|
| **Q64.1** | 1 | 2 | 99 | **Q64.1** |

| | | | | |
|---|---|---|---|---|
| during your first year of life | | | | خلال أول عام من حياتك |
| **Q64.2**<br>when you were aged 1 to 4 years | 1 | 2 | 99 | **Q64.2**<br>عندما كان عمرك يتراوح بين 1 إلى 4 أعوام |
| **Q64.3**<br>when you were aged 5-15 years | 1 | 2 | 99 | **Q64.3**<br>عندما كان عمرك يتراوح بين 5 إلى 15 عاماً. |

65. Was there a dog in your home? هل كان لديك كلب فى منزلك؟ .65

| | Yes<br>نعم | No<br>لا | DK<br>لا أعرف | |
|---|---|---|---|---|
| **Q65.1**<br>during your first year of life | 1 | 2 | 99 | **Q65.1**<br>خلال أول عام من حياتك |
| **Q65.2**<br>when you were aged 1 to 4 years | 1 | 2 | 99 | **Q65.2**<br>عندما كان عمرك يتراوح بين 1 إلى 4 أعوام |
| **Q65.3**<br>when you were aged 5-15 years | 1 | 2 | 99 | **Q65.3**<br>عندما كان عمرك يتراوح بين 5 إلى 15 عاماً. |

Q 66. Was there a bird in your home? هل كان لديك طيور فى منزلك؟ Q.66

| | Yes<br>نعم | No<br>لا | DK<br>لا أعرف | |
|---|---|---|---|---|
| **Q66.1.**<br>during your first year of life | 1 | 2 | 99 | **Q66.1**<br>خلال أول عام من حياتك |
| **Q66.2**<br>when you were aged 1 to 4 years | 1 | 2 | 99 | **Q66.2**<br>عندما كان عمرك يتراوح بين 1 إلى 4 أعوام |
| **Q66.3**<br>when you were aged 5-15 years | 1 | 2 | 99 | **Q66.3**<br>عندما كان عمرك يتراوح بين 5 إلى 15 عاماً. |

Q67. What term best describes the place you lived most of the time when you were under the age of five years? TICK ONE BOX ONLY

ما أفضل مصطلح يصف المكان الذى كنت تعيش فيه أغلب الوقت عندما كان عمرك أقل من 5 أعوام؟ ضع علامة على مربع واحد فقط Q.67

| | | |
|---|---|---|
| a) farm | 1 | a) مزرعة |
| b) village in a rural area | 2 | b) قرية فى منطقة ريفية |
| c) small town | 3 | c) بلدة صغيرة |
| d) suburb of a city | 4 | d) ضاحية لمدينة |
| e) inner city | 5 | e) داخل المدينة |

Q68. When you are near animals, such as cats, dogs or horses, do you ever

Q .68 عند وجودك بالقرب من الحيوانات، مثل القطط، الكلاب أو الخيول، هل:

| | Yes<br>نعم | No<br>لا | |
|---|---|---|---|
| **Q 68.1**<br><br>start to cough? | 1 | 2 | **Q 68.1**<br>بدأت فى السعال؟ |
| **Q 68.2**<br><br>start to wheeze? | 1 | 2 | **Q 68.2**<br>بدأت فى الشعور بأزيز؟ |
| **Q 68.3**<br><br>get a feeling of tightness in your chest? | 1 | 2 | **Q68.3**<br>بدأت فى الشعور بضيق فى صدرك؟ |
| **Q 68.4**<br><br>start to feel short of breath? | 1 | 2 | Q 68.4<br>بدأت فى الشعور بضيق فى التنفس؟ |
| **Q 68.5**<br><br>get a runny or stuffy nose or start to sneeze? | 1 | 2 | Q 68.5<br>أصابك رشح أو انسداد فى الأنف أو بدأت فى العطس؟ |
| **Q 68.6**<br><br>get itchy or watering eyes? | 1 | 2 | Q 68.6<br>أصابك حك أو دموع بالعين؟ |

Q69. When you are in a dusty part of the house, or near pillows or duvet s do you ever

Q.69 عند وجودك فى جزء به غبار من منزلك، أو بالقرب من الوسائد أو القماش المخملى هل:

| | Yes<br>نعم | No<br>لا | |
|---|---|---|---|
| **Q69.1**<br><br>start to cough? | 1 | 2 | **Q 69.1**<br>بدأت فى السعال؟ |
| **Q69.2** | 1 | 2 | **Q 69.2**<br>بدأت فى الشعور بأزيز؟ |

| | | | |
|---|---|---|---|
| start to wheeze? | | | |
| **Q 69.3**<br><br>get a feeling of tightness in your chest? | 1 | 2 | **Q 69.3**<br>بدأت فى الشعور بضيق فى صدرك؟ |
| **Q 69.4**<br><br>start to feel short of breath? | 1 | 2 | **Q 69.4**<br>بدأت فى الشعور بضيق فى التنفس؟ |
| **Q 69.5**<br><br>get a runny or stuffy nose or start to sneeze? | 1 | 2 | **Q 69.5**<br>أصابك رشح أو انسداد فى الأنف أو بدأت فى العطس؟ |
| **Q 69.6**<br><br>get itchy or watering eyes? | 1 | 2 | **Q 69.6**<br>أصابك حك أو دموع بالعين؟ |

Q70. When you are near trees, grass or flowers, or when there is a lot of pollen about, do you ever

70. Qعندما تكون بالقرب من أشجار أو أعشاب أو زهور ويكون هناك الكثير من حبوب اللقاح، هل حدث لك أن .....من قبل

| | Yes<br>نعم | No<br>لا | |
|---|---|---|---|
| **Q70.1**<br><br>start to cough? | 1 | 2 | Q**70.1**<br>بدأت تسعل؟ |
| **Q70.2**<br><br>start to wheeze? | 1 | 2 | بدأت **Q70.2**<br>تشعر بأزيز ؟ |
| **Q70.3**<br><br>get a feeling of tightness in your chest? | 1 | 2 | **Q70.3**<br><br>تشعر بضيق في صدرك؟ |
| **Q70.4**<br><br>start to feel short of breath? | 1 | 2 | Q**70.4**<br><br>بدأت تشعر بضيق في التنفس؟ |
| **Q70.5**<br><br>get a runny or stuffy nose or start to sneeze? | 1 | 2 | Q70.5<br><br>عانيت من رشح في الأنفأو انسداد أو بدأت تعطس؟ |
| Q70.6<br><br>get itchy or watering eyes? | 1 | 2 | Q70.6<br><br>تشعر بحكة أو بدموع في العين؟ |

IF 'YES' TO ANY OF THE ABOVE:
Then ask 70.7.1.4 otherwise go to Q71

إذا كانت الإجابة ‘نعم‘ لأي من الإجابات السابقة:
سأل 70.7.1.4 أخرى انتقل إلى Q71

Q70.7.1-4 Which time of year does this happen?

Q70.7.1-4 في أي وقت من السنة يحدث هذا؟

| | Yes<br>نعم | No<br>لا | |
|---|---|---|---|
| **Q70.7.1**<br>**winter** | 1 | 2 | **Q70.7.1**<br>الشتاء |
| **Q70.7.2**<br>**spring** | 1 | 2 | **Q70.7.2**<br>الربيع |
| **Q70.7.3**<br>**summer** | 1 | 2 | **Q70.7.3**<br>الصيف |
| **Q70.7.4**<br>**autumn** | 1 | 2 | **Q70.7.4**<br>الخريف |

Q71. How often do you eat pre-packaged food, such as tinned food or pre-prepared frozen meals? TICK ONE BOX ONLY

Q71. ما هو معدل تناولك أطعمة محفوظة مثل الطعام المعلب أو الوجبات المثلجة سابقة التحضير؟قم بوضع علامة على مربع واحد فقط

| | | |
|---|---|---|
| a) every day or most days | 1 | a) يومياً أو معظم الأيام |
| b) at least once a week | 2 | b) على الأقل مرة في الأسبوع |
| c) less than once a week | 3 | c) أقل من مرة في الأسبوع |

Q72. Do you take snacks between meals?

Q72. هل تتناول وجبات خفيفة بين الوجبات الرئيسية؟

| | | |
|---|---|---|
| Yes ( Go to 72.1.1-3 ) | 1 | نعم (انتقل إلى 72.1.1-3) |
| No ( Go to 73 ) | 2 | لا ( انتقل إلى 73) |

Q72.1.1-3 Which of the following would you have as a snack at least once a week?

Q 72.1.1-3 أي من الأطعمة التالية تتناولها كوجبات خفيفة على الأقل مرة في الأسبوع؟

| | Yes<br>نعم | No<br>لا | |
|---|---|---|---|

| | | | |
|---|---|---|---|
| **Q72.1.1**<br><br>savory biscuits or crisps | 1 | 2 | **Q 72.1.1**<br>بسكويت مملح أو مقرمشات |
| **Q72.1.2**<br><br>sweets, chocolates or sweet biscuits | 1 | 2 | **Q 72.1.2**<br>حلوى أو شوكولاتة أو بسكويت محلى |
| Q72.1.3<br><br>fruit or vegetables | 1 | 2 | **Q 72.1.3**<br>فواكهة أو خضروات |

Q73. Have you ever had an illness or trouble caused by eating a particular food or foods?

Q73 هل سبق أن أصبت بمرض أو عانيت من مشكلة نتيجة تناولك طعام أو أطعمة معينة؟

| | | |
|---|---|---|
| Yes ( Go to 73.1 ) | 1 | نعم (انتقل إلى 73.1) |
| No ( Go to 74 ) | 2 | لا ( انتقل إلى 74) |

Q73.1 Have you nearly always had the same illness or trouble after eating this type of food?

Q73.1 هل تُصاب عادةً بنفس المرض أو تعاني من نفس المشكلة بعد تناول نفس نوع الطعام؟

| | | |
|---|---|---|
| Yes ( Go to 73.1.1 ) | 1 | نعم (انتقل إلى 73.1.1) |
| No ( Go to 74 ) | 2 | لا ( انتقل إلى 74) |

Q73.1.1 What type of food was this? [List up to 3]

Q73.1.1 ماذا كان نوع هذا الطعام؟ [ضع حتى 3 أنواع في قائمة]

| | |
|---|---|
| Food 1<br><br>الطعام 1 | |
| Food 2<br><br>الطعام 2 | |
| Food 3<br><br>الطعام 3 | |

Q73.1.2.1-6 : Did this illness or food allergy include

Q73.1.2.1-6 هل صاحب هذا المرض أو الحساسية لبعض الأطعمة:

| | Yes<br>نعم | No<br>لا | |
|---|---|---|---|
| **Q73.1.2.1** | 1 | 2 | **Q73.1.2.1**<br>طفح جلدي أو حكة في الجلد؟ |

| | | | |
|---|---|---|---|
| a rash or itchy skin? | | | |
| **Q73.1.2.2**<br>diarrhoea or vomiting? | 1 | 2 | **Q73.1.2.2**<br>اسهال أو قيء؟ |
| **Q73.1.2.3**<br>runny or stuffy nose? | 1 | 2 | **Q73.1.2.3**<br>رشح أو انسداد في الأنف؟ |
| **Q73.1.2.4**<br>severe headaches? | 1 | 2 | **Q73.1.2.4**<br>صداع حاد؟ |
| **Q73.1.2.5**<br>breathlessness? | 1 | 2 | **Q73.1.2.5**<br>صعوبة في التنفس؟ |
| **Q73.1.2.6**<br>other: ______________________________ _ | | | **Q73.1.2.6**<br>أخرى: ____________________________ __________ |

Q74. Have you ever smoked for as long as a year?

هل سبق وكنت تدخن لمدة سنة؟ Q74.

['YES' means at least 20 packs of cigarettes or 12 oz (360 grams) of tobacco in a lifetime, or at least one cigarette per day or one cigar a week for one year]

[إذا كانت ‘نعم‘ تعني على الأقل 20 علبة سجائر أو 12 أوقيه (360 جرام) من التبغ في العمر، أو على الأقل سيجارة واحدة في اليوم أو سيجار واحد في الأسبوع لمدة سنة]

| | | |
|---|---|---|
| Yes ( Go to 74.1 ) | 1 | نعم (انتقل إلى 74.1) |
| No ( Go to 75 ) | 2 | لا ( انتقل إلى 75) |

Q74.1 How old were you when you started smoking?

كم كان عمرك عندما بدأت تدخن؟ Q74.1

| | | |
|---|---|---|
| Age (years) | | العمر (سنة) |

Q74.2 Do you now smoke, as of one month ago?

هل تدخن حالياً، أي منذ شهر مثلاً؟ Q74.2

| | | |
|---|---|---|
| Yes ( Go to 74.2.1-4 ) | 1 | نعم (انتقل إلى 74.2.1-4) |
| No ( Go to 74.3 ) | 2 | لا ( انتقل إلى 74.3) |

Q74.2.1-4 How much do you now smoke on average?

Q74.2.1-4 ما هو معدل تدخينك حالياً تقريباً؟

| | Number<br>العدد | |
|---|---|---|
| Q74.2.1<br>number of cigarettes per day | | Q74.2.1<br>عدد السجائر في اليوم |
| Q74.2.2<br>number of cigarillos per day | | Q74.2.2<br>عدد السيجار الصغير في اليوم |
| Q74.2.3<br>number of cigars a week | | Q74.2.3<br>عدد السيجار في الأسبوع |
| Q74.2.4<br>pipe tobacco in a) ounces / week | | Q74.2.4<br>التبغ المخصص للغليون (بايب) بالأوقية/ في الأسبوع |
| Q74.2.5<br>pipe tobacco in a) grams / week | | Q74.2.5<br>التبغ المخصص للغليون (بايب) بالجرام/ في الأسبوع |

Q74.3 Have you stopped or cut down smoking?

Q74.3 هل توقفت أوانتقطعت عن التدخين ؟

| | | |
|---|---|---|
| Yes ( Go to 74.3.1 ) | 1 | نعم (انتقل إلى 74.3.1) |
| No ( Go to 74.4 ) | 2 | لا ( انتقل إلى 74.4) |

Q74.3.1 how old were you when you stopped or cut down smoking?

Q74.3.1 كم كان عمرك عندما توقفت عن التدخين أو انقطعت عنه؟

| | | |
|---|---|---|
| Age (Years) | | العمر (سنة) |

74.3.2.1-4 on average of the entire time you smoked, before you stopped or cut down, how much did you smoke?

74.3.2.1-4 بالنسبة لمتوسط الوقت الذي قمت فيه بالتدخين، قبل التوقف عن التدخين أو الانقطاع عنه، ما هي المدة التي كنت تدخن فيها؟

| Yes | Number<br>العدد | نعم |
|---|---|---|
| **Q74.2.1**<br>number of cigarettes per day | | **Q74.2.1**<br>عدد السجائر في اليوم |
| **Q74.2.2**<br>number of cigarillos per day | | **Q74.2.2**<br>عدد السيجار الصغير في اليوم |
| **Q74.2.3**<br>number of cigars a week | | **Q74.2.3**<br>عدد السيجار في الأسبوع |
| **Q74.2.4**<br>pipe tobacco in a) ounces / week | | **Q74.2.4**<br>التبغ المخصص للغليون (بايب) بالأوقية/ في الأسبوع |
| **Q74.2.5**<br>pipe tobacco in a) grams / week | | **Q74.2.5**<br>التبغ المخصص للغليون (بايب) بالجرام/ في الأسبوع |

74.4 Do you or did you inhale the smoke?

74.4 هل تستنشق أو استنشقت الدخان؟

| | | |
|---|---|---|
| Yes | 1 | نعم |
| No | 2 | لا |

75. Have you been regularly exposed to tobacco smoke in the last 12 months? ['Regularly' means on most days or nights]

75. هل كنت تتعرض لدخان التبغ بشكل منتظم في الأثنى عشرة شهر الماضية؟ [‘بشكل منتظم‘ تعني في معظم الأيام أو الليالي]

| | | |
|---|---|---|
| Yes ( Go to 75.1 ) | 1 | نعم (انتقل إلى 75.1) |
| No ( Go to 76 ) | 2 | لا ( انتقل إلى 76) |

75.1. Not counting yourself, how many people in your household smoke regularly?

75.1. لا تحسب نفسك، كم عدد الأشخاص الذين يدخنون بشكل منتظم في أسرتك؟

| | | |
|---|---|---|
| Number of people | | عدد الأشخاص |

75.2 Do people smoke regularly in the room where you work?

75.2 هل يدخن الناس عادة في الحجرة التي تعمل بها؟

| | | |
|---|---|---|
| Yes | 1 | نعم |
| No | 2 | لا |

75.3 How many hours per day are you exposed to other people's tobacco smoke?

75.3 كم عدد الساعات التي تتعرض فيها لدخان سجائر بالأشخاص الأخرون يومياً؟

| | | |
|---|---|---|
| Hours per day | | عدد الساعات يومياً |

75.4 Please provide more information. How many hours per day, are you exposed to other peoples tobacco smoke in the following locations?

75.4 من فضلك أخبرنا بمعلومات أكثر. كم عدد الساعات التي تعرضت فيها لدخان سجائر الأشخاص الأخرون يومياً في الأماكن التالية؟

| | Number of hours<br>عدد الساعات | |
|---|---|---|
| at home | | في المنزل |
| at workplace | | في مكان العمل |
| in bars, restaurants, cinemas or similar social settings | | في الحانات أو المطاعم او دور العرض السينمائي أو أماكن اجتماعية مشابهة |
| elsewhere | | أماكن أخرى |

76. Have you used any inhaled medicines to help your breathing at any time in the last ***12 months***?

76. هل قمت باستخدام أدوية استنشاق لتساعد على تنفسك في أي وقت في **الأثنى عشرة شهراً** الماضية؟

| | | |
|---|---|---|
| Yes ( Go to 76.0 ) | 1 | نعم (انتقل إلى 76.0) |
| No ( Go to 77 ) | 2 | لا ( انتقل إلى 77) |

Q 76-0 Which of the following have you used in the last 12 months?

Q76-0 أي من الأتي استخدمت في الاثنى عشرة شهراً الماضية؟

| | Yes<br>نعم | No<br>لا | |
|---|---|---|---|
| **Q76.1**<br>Short-acting beta-2-agonist inhalers | 1 | 2 | **Q76.1**<br>أدوية الاستنشاق من نوع محفزات البيتا 2 قصيرة المفعول |
| **Q76.2**<br>Long-acting beta-2-agnonist inhalers | 1 | 2 | **Q76.2**<br>أدوية الاستنشاق من نوع محفزات البيتا 2 طويلة المفعول |
| **Q76.3**<br>Anti-muscarinic/ anti-cholinergic inhalers | 1 | 2 | **Q76.4**<br>أدوية الاستنشاق من نوع مضاد المسكارين/ الأدوية المضادة للكولين |
| **Q76.4**<br>Inhaled steroids | 1 | 2 | Q76.5<br>الاسترويد المستنشق |
| **Q76.5**<br>Inhaled cromoglycate/ nedocromil | 1 | 2 | Q76.6<br>كروموجلايكيت المستنشق/ نيدوكروميل |

Q76.1.1 If "YES" to 76.1, then which one?
Show card image and medicine list

Q76.1.1 إذا كانت الإجابة "نعم" على 76.1، أي من التالي؟ اعرض البطاقة بالصورة وقائمة الدواء

| | | Yes<br>نعم | No<br>لا | |
|---|---|---|---|---|
| Asthalin | 1 | 1 | 2 | استالين |
| Ventolin | 2 | 1 | 2 | فينتولين |
| Derihaler | 3 | 1 | 2 | ديريهالار |
| Vent easeecaps | 4 | 1 | 2 | فينت أيسيكابس |
| Other | 5 | 1 | 2 | أخرى |
| Other | 6 | 1 | 2 | أخرى |

76.1.2 What type of inhaler do you use? TICK ONE BOX ONLY

76.1.2 ما هو نوع دواء الاستنشاق الذي تستخدمه؟ ضع علامة على مربع واحد فقط

| | | |
|---|---|---|
| MDI | 1 | البخاخ قياسي الجرعة |
| Dry powder | 2 | بودرة جافة |
| Nebuliser | 3 | مُبَخِر |
| Not known | 4 | لا أعرف |

76.1.3. What is the dose per puff (in micrograms)?

76.1.3. ما هي الجرعة في النفخة الواحدة (بالميكروجرام)

| | | |
|---|---|---|
| Dose (mg) | | الجرعة (ميكروجرام) |

Q76.1.4. In the last 3 months, how have you used them:TICK ONE BOX ONLY

76.1.4. في الثلاثة أشهر الماضية، كم مرة استخدمتهم:ضع علامة واحدة على مربع واحد فقط

| | | |
|---|---|---|
| a) when needed ( Go to Q76.1.5) | 1 | a) عند الحاجة إليه (انتقل إلى Q 76.1.5) |
| b) in short courses ( Go to Q76.1.6 ) | 2 | b) خلال فترات قصيرة ( انتقل إلى Q 76.1.6) |
| c) continuously ( Go to Q76.1.9 ) | 3 | c) باستمرار ( انتقل إلى Q 76.1.9) |
| d) not at all ( Go to 76.2) | 4 | d) لا استخدمه على الإطلاق (انتقل إلىQ 76.2) |

Q76.1.5 Number of puffs per month

76.1.5 عدد النفخات في الشهر

| | | |
|---|---|---|
| Number of puffs (per month) | | عدد النفخات (في الشهر) |

| | | |
|---|---|---|
| **Q76.1.6**<br>number of courses | | **Q76.1.6**<br>عدد الفترات |
| **Q76.1.7**<br>number of puffs per day | | **Q76.1.7**<br>عدد النفخات في اليوم |
| **Q76.1.8**<br>average number of days per month | | **Q76.1.8**<br>متوسط عدد الأيام في الشهر |

76.1.9 number of puffs per day

76.1.9 عدد النفخات في اليوم

| | | |
|---|---|---|
| Number of puffs (per day) | | عدد النفخات (في اليوم) |

76.2.1 IF YES TO 76.2 (Long-acting beta-2-agnonist inhalers), which one? Image and list Show card

76.2.1 إذا كانت الإجابة "نعم" على 76.2 (أدوية الاستنشاق من نوع محفزات البيتا 2 طويلة المفعول)، أي من التالي؟ الصورة والقائمة اعرض البطاقة

| | | |
|---|---|---|
| Serevent | 1 | سيريفت |
| Foradil | 2 | فوراديل |
| Duova | 3 | دوفا |
| Fortec | 4 | فورتك |
| Derisone | 5 | ديريسون |
| Seretide | 6 | سيريتايد |
| Symbicort | 7 | سيمبيكورت |
| Foracort | 8 | فوراكورت |
| Seroflo | 9 | سيروفلو |
| Aerocort | 10 | أيروكورت |
| Combimist | 11 | كومبيميست |
| Esiflo | 12 | اسيفلو |
| Vent FB | 13 | فينت اف بيه |
| Vent SF | 14 | فينت اس اف |
| Other | 15 | أخرى |
| Other | 16 | أخرى |

76.2.2 What type of inhaler do you use? TICK ONE BOX ONLY ( Image show card)

76.2.2 ما هو نوع دواء الاستنشاق الذي تستخدمه؟ ضع علامة على مربع واحد فقط (الصورة اعرض البطاقة)

| | | |
|---|---|---|
| MDI | 1 | البخاخ قياسي الجرعة |
| Dry powder | 2 | بودرة جافة |
| Nebuliser | 3 | مُبَخِر |
| Not known | 4 | لا أعرف |

76.2.3. What is the dose per puff (in micrograms)?

76.2.3. ما هي الجرعة في النفخة الواحدة (بالميكروجرام)

| | | |
|---|---|---|
| Dose (mg) | | الجرعة (ميكروجرام) |

76.2.4. In the last 3 months, how have you used them:TICK ONE BOX ONLY

76.2.4. في الثلاثة أشهر الماضية، كم مرة استخدمتهم: ضع علامة واحدة على مربع واحد فقط

| | | |
|---|---|---|
| a) when needed | 1 | a) عند الحاجة إليه |
| b) in short courses | 2 | b) خلال فترات قصيرة |

| | | |
|---|---|---|
| c) continuously | 3 | c) باستمرار |
| d) not at all ( Go to 76.3.1) | 4 | d) لا استخدمه على الإطلاق (انتقل إلى 76.3.1) |

**<u>If answer to 76.2.4 is when needed:</u>** **<u>إذا تم إختيار إجابة "عند الحاجة إليه" في سؤال 76.2.4 :</u>**

76.2.5 Number of puffs per month 76.2.5 عدد النفخات في الشهر

| | | |
|---|---|---|
| Number of puffs (per month) | | عدد النفخات (في الشهر) |

**<u>If answer to 76.2.4 is in short courses</u>** ***<u>إذا تم اختيار إجابة " خلال فترات قصيرة" في سؤال 76.2.4</u>***

| | | |
|---|---|---|
| **Q76.2.6**<br>number of courses | | **Q76.2.6**<br>عدد الفترات |
| Q76.2.7<br>number of puffs per day | | **Q76.2.7**<br>عدد النفخات في اليوم |
| Q76.2.8<br>average number of days per month | | **Q76.2.8**<br>متوسط عدد الأيام في الشهر |

***<u>If answer to 76.2.4 4 is continuously</u>*** ***<u>إذا تم اختيار إجابة "باستمرار" في سؤال 76.2.4</u>***

76.2.9 number of puffs per day 76.2.9عدد النفخات في اليوم

| | | |
|---|---|---|
| Number of puffs (per day) | | عدد النفخات (في اليوم) |

76.3.1 If “YES” to Q76.3(Anti-muscarinic/ anti-cholinergic inhalers) , then which one? Image and list Show cards

76.3.1 إذا كانت الإجابة "نعم" علىQ76.3 (أدوية الاستنشاق من نوع مضاد المسكارين/ الأدوية المضادة للكولين ) أي من التالي؟ الصورة والقائمة اعرض البطاقات

| | | |
|---|---|---|
| Tiova | 1 | تي أوفا |
| Spiriva | 2 | سبيريفيا |
| Ipravent DP caps | 3 | إبرافيت دي بي كبسولات |
| Ipramist | 4 | إبراميست |

| | | |
|---|---|---|
| Other | 5 | أخرى |
| Other | 6 | أخرى |

76.3.2 What type of inhaler do you use? TICK ONE BOX ONLY ( Image Show cads)

76.3.2 ما هو نوع دواء الاستنشاق الذي تستخدمه؟ ضع علامة على مربع واحد فقط (الصورة اعرض البطاقات)

| | | |
|---|---|---|
| MDI | 1 | البخاخ قياسي الجرعة |
| Dry powder | 2 | بودرة جافة |
| Nebuliser | 3 | مُبَخِر |
| Not known | 4 | لا أعرف |

76.3.3. What is the dose per puff (in micrograms)?

76.3.3. ما هي الجرعة في النفخة الواحدة (بالميكروجرام)

| | | |
|---|---|---|
| Dose (mg) | | الجرعة (ميكروجرام) |

76.3.4. In the last 3 months, how have you used them:TICK ONE BOX ONLY

76.3.4. في الثلاثة أشهر الماضية، كم مرة استخدمتهم: ضع علامة واحدة على مربع واحد فقط

| | | |
|---|---|---|
| a) when needed | 1 | a) عند الحاجة إليه |
| b) in short courses | 2 | b) خلال فترات قصيرة |
| c) continuously ( Go to 76.4.1) | 3 | c) باستمرار (انتقل إلىQ 76.4.1) |
| d) not at all ( Go to 76.4.1) | 4 | d) لا استخدمه على الإطلاق (انتقل إلىQ 76.4.1) |

**<u>If answer to 76.3.4 is when needed:</u>**

**<u>إذا تم إختيار إجابة "عند الحاجة إليه" فى سؤال 76.3.4 :</u>**

76.3.5 Number of puffs per month

76.3.5عدد النفخات في الشهر

| | | |
|---|---|---|
| Number of puffs (per month) | | عدد النفخات (في الشهر) |

***<u>If answer to 76.3.4 is in short courses</u>***

***<u>إذا تم اختيار إجابة "خلال فترات قصيرة" فى سؤال 76.3.4</u>***

| | | |
|---|---|---|
| **Q76.3.6**<br>number of courses | | **Q76.4.6**<br>عدد الفترات |
| **Q76.3.7**<br>number of puffs per day | | **Q76.4.7**<br>عدد النفخات في اليوم |
| **Q76.3.8**<br>average number of days per month | | **Q76.4.8**<br>متوسط عدد الأيام في الشهر |

| | | |
|---|---|---|
| 76.3.9<br>number of puffs per day | | 76.4.9<br>عدد النفخات في اليوم |

76.4.1 If “YES” to Q 76.4 (Inhaled Steroids), then which one? SHOW CARDS

76.4.1إذا كانت الإجابة "نعم" على Q 76.4 (الاسترويد المستنشق )، أي من التالي؟ اعرض البطاقات

| | | |
|---|---|---|
| Alvesco | 1 | ألفيسكو |
| Flixotide | 2 | فليكسوتايد |
| Pulmicort | 3 | بلميكورت |
| Budecort | 4 | بوديكورت |
| Asmacort | 5 | أسماكورت |
| Flohale | 6 | فلوهال |
| Other | 7 | أخرى |
| Other | 8 | أخرى |

76.4.2 What type of inhaler do you use? TICK ONE BOX ONLY ( Image Show cards)

76.4.2 ما هو نوع دواء الاستنشاق الذي تستخدمه؟ ضع علامة على مربع واحد فقط (_الصورة اعرض البطاقات)

| | | |
|---|---|---|
| MDI | 1 | البخاخ قياسي الجرعة |
| Dry powder | 2 | بودرة جافة |
| Nebuliser | 3 | مُبَخِر |
| Not known | 4 | لا أعرف |

76.4.3. What is the dose per puff (in micrograms)?

76.4.3. ما هي الجرعة في النفخة الواحدة (بالميكروجرام)

| | | |
|---|---|---|
| Dose (mg) | | الجرعة (ميكروجرام) |

76.4.4. In the last 3 months, how have you used them:TICK ONE BOX ONLY

76.4.4. في الثلاثة أشهر الماضية، كم مرة استخدمتهم:ضع علامة واحدة على مربع واحد فقط

| | | |
|---|---|---|
| a) when needed | 1 | a) عند الحاجة إليه |
| b) in short courses | 2 | b) خلال فترات قصيرة |
| c) continuously | 3 | c) باستمرار |
| d) not at all ( Go to 76.6) | 4 | d) لا استخدمه على الإطلاق (انتقل إلى 76.6) |

**If answer to 76.4.4 is when needed:**

**إذا تم إختيار إجابة "عند الحاجة إليه" في سؤال 76.4.4 :**

76.5.5 number of puffs per month 76.5.5عدد النفخات في الشهر

| Number of puffs (per month) | | عدد النفخات (في الشهر) |
|---|---|---|

***If answer to 76.4.4 is in short courses*** ***إذا تم اختيار إجابة "خلال فترات قصيرة" في سؤال 76.4.4***

| Q76.5.6<br>number of courses | | **Q76.5.6**<br>عدد الفترات |
|---|---|---|
| Q76.5.7<br>number of puffs per day | | **Q76.5.7**<br>عدد النفخات في اليوم |
| Q76.5.8<br>average number of days per month | | **Q76.5.8**<br>متوسط عدد الأيام في الشهر |

***If answer to 76.5.4 is continuously*** ***إذا تم اختيار إجابة " باستمرار" في سؤال 76.5.4***

76.5.9 number of puffs per day 76.5.9عدد النفخات في اليوم

| Number of puffs (per day) | | عدد النفخات (في اليوم) |
|---|---|---|

76.6 Inhaled cromoglycate/ nedocromil 76.6 كروموجلايكيت المستنشق/ نيدوكروميل

76.6. If “YES” to Q76.5 (Inhaled cromoglycate/ nedocromil), then which one? SHOW CARDS

76.6.1إذا كانت الإجابة "نعم" على Q76.5 (كروموجلايكيت المستنشق/ نيدوكروميل) أي من التالي؟ اعرض البطاقات

| | | |
|---|---|---|
| Cromolyn | 1 | كرومولين |
| Other | 2 | أخرى |
| Other | 3 | أخرى |

76.6.2. What is the dose per puff (in micrograms)? 76.6.2. ما هي الجرعة في النفخة الواحدة (بالميكروجرام)

| Dose (mg) | | الجرعة (ميكروجرام) |
|---|---|---|

76.6.3. In the last 3 months, how have you used them:TICK ONE BOX ONLY

76.6.3. في الثلاثة أشهر الماضية، كم مرة استخدمتهم:
ضع علامة واحدة على مربع واحد فقط

| | | |
|---|---|---|
| a) when needed | 1 | عند الحاجة إليه (a |
| b) in short courses | 2 | خلال فترات قصيرة (b |
| c) continuously | 3 | باستمرار (c |
| d) not at all ( Go to 77) | 4 | لا استخدمه على الإطلاق (انتقل إلى77 Q) (d |

**<u>If answer to 76.6.3 is when needed</u>** **<u>إذا تم اختيار إجابة "عند الحاجة إليه" في سؤال 76.6.3</u>**

76.6.4 number of puffs per month

76.6.4عدد النفخات في الشهر

| | | |
|---|---|---|
| Number of puffs (per day) | | عدد النفخات (في اليوم) |

***<u>If answer to 76.6.3 is in short courses</u>*** ***<u>إذا تم اختيار إجابة "خلال فترات قصيرة" في سؤال 76.6.3</u>***

| | | |
|---|---|---|
| 76.6.5 number of courses | | 76.6.5 عدد الفترات |
| 76.6.6 number of puffs per day | | 76.6.5 عدد النفخات في اليوم |
| 76.6.7 average number of days per month | | 76.6.5 متوسط عدد الأيام في الشهر |

***<u>If answer to 76.6.3 is continuously</u>*** ***<u>إذا تم اختيار إجابة "باستمرار" في سؤال 76.6.3</u>***

76.6.8 number of puffs per day

76.6.8عدد النفخات في اليوم

| | | |
|---|---|---|
| Number of puffs (per day) | | عدد النفخات (في اليوم) |

77. Have you used any pills, capsules, tablets or medicines, other than inhaled medicines, to help your breathing at any time in the last 12 months?

77. هل استخدمت أقراص أو كبسولات أو أدوية غير الأدوية المستنشقة لمساعدتك على التنفس في أي وقت في الأثنى عشرة شهراً الماضية؟

| | | |
|---|---|---|
| Yes ( Go to Q77.0) | 1 | نعم (انتقل إلى Q77.0) |
| No ( Go to 78) | 2 | لا (انتقل إلى 78) |

Q77.0 Which of the following have you used in the last 12 months?

Q 77.0أي من الأدوية التالية استخدمت من قبل في الأثنى عشرة شهراً الماضية؟

| | Yes<br>نعم | No<br>لا | |
|---|---|---|---|
| **Q77.1**<br>oral beta-2-agnonists | 1 | 2 | **Q77.1**<br>معززات البيتا 2 التي يتم تناولها عن طريق الفم |
| **Q77.2**<br>oral methylxanthines | 1 | 2 | **Q77.2**<br>الميثيل زانثينات |
| Q77.3<br>oral steroids | 1 | 2 | **Q77.3**<br>ستيرويد يتم تناوله عن طريق الفم |
| Q77.4<br>oral leukotrienes | 1 | 2 | **Q77.4**<br>مركبات الليكوترين |
| Q77.5<br>ketotifen | 1 | 2 | 77.5 كيتوتيفن |

77.1.1 If "YES" used to Q77.1 (oral beta-2-agnonists), then which one? SHOW CARDS

77.1 إذا كانت الإجابة "نعم" على سؤال Q77.1 (معززات البيتا 2 التي يتم تناولها عن طريق الفم)، أي من التالي؟ اعرض البطاقات

| | | |
|---|---|---|
| Asthalin SR | 1 | أسثالين اس ار |
| Bambudil | 2 | بامبوديل |
| Ventoin CR | 3 | فينتولين سي ار |
| Bricanyl Durules | 4 | بريكانيل دورولز |
| Other | 5 | أخرى |
| Other | 6 | أخرى |

77.1.2 what dose of tablet

77.1.2 ما هي الجرعة بالأقراص؟

| Dose | | الجرعة |
|---|---|---|

77.1.3. In the last 3 months, how have you used them:TICK ONE BOX ONLY

77.1.3.في الثلاثة أشهر الماضية، كم مرة استخدمتهم: ضع علامة واحدة على مربع واحد فقط

| | | |
|---|---|---|
| a) when needed | 1 | a) عند الحاجة إليه |
| b) in short courses | 2 | b) خلال فترات قصيرة |
| c) continuously | 3 | c) باستمرار |
| d) not at all ( Go to 77.2.1) | 4 | d) لا استخدمه على الإطلاق (انتقل إلى77.2.1) |

**If answer to 77.1.3 is needed** **إذا تم اختيار إجابة "عند الحاجة إليه" في سؤال 77.1.3**

77.1.4 number of tablets per month 77.1.4 عدد الأقراص في الشهر

| | | |
|---|---|---|
| Number of tablets (month) | | عدد الأقراص (في الشهر) |

***If answer to 77.1.3 is in short courses*** ***إذا تم اختيار إجابة "خلال فترات قصيرة" في سؤال 77.1.3***

| | | |
|---|---|---|
| 77.1.5<br>number of courses | | 77.1.5<br>عدد الفترات |
| 77.1.6<br>tablets per day | | 77.1.6<br>عدد النفخات في اليوم |
| 77.1.7<br>average number of days per month | | 77.1.7<br>متوسط عدد الأيام في الشهر |
| | | |

***If answer to 77.1.3 is continuously*** ***إذا تم اختيار إجابة "باستمرار" في سؤال 77.1.3***

77.1.8 tablets per day 77.1.8 الأقراص فى اليوم الواحد

Number of tablets (day)----------

عدد الأقراص (اليوم)-------------

77.2.1 If "YES" used to Q77.2 (oral methylxanthines), then which one? SHOW CARDS

77.2.1 إذا كانت الإجابة "نعم" على Q77.2 (الميثيل زانثينات التى يتم تناولها عن طريق الفم) أي من التالي؟ اعرض البطاقات

| | | |
|---|---|---|
| Neulin | 1 | نيولين |
| quibron | 2 | كيبرون |
| Deriphylline OD | 3 | دريفيلين OD |
| theoasthalin | 4 | ثيو ثلين |
| Daxas | 5 | داكساس |
| Unicontin | 6 | يونيكونتين |
| theobric SR | 7 | ثيوبرك SR |
| Duralyn CR | 8 | ديورالين CR |
| Durasal | 9 | ديوراسال |
| synasma | 10 | سيناسما |
| Other | 11 | أخرى |
| Other | 12 | أخرى |

77.2.2 what dose of tablet — 77.2.2 ما هى جرعة الأقراص؟

Dose -------------- الجرعة

77.2.3. In the last 3 months, how have you used them: TICK ONE BOX ONLY — 77.2.3 كيف استخدمتهم خلال الثلاثة أشهر الماضية: ضع علامة واحدة على مربع واحد فقط

| | | |
|---|---|---|
| a) when needed | 1 | عند الحاجة (a |
| b) in short courses | 2 | خلال فترات قصيرة (b |
| c) continuously | 3 | باستمرار (c |
| d) not at all ( Go to 77.3.1) | 4 | لا استخدمهم على الإطلاق (انتقل إلى Q 77.3.1) (d |

**If answer to 77.2.3 is when needed:** — **إذا كانت الإجابة على 77.2.3 هى "عند الحاجة إليها": سيكون**

77.2.4 number of tablets per month — 77.2.4 عدد الأقراص فى الشهر

| | | |
|---|---|---|
| Number of tablets (month) | | عدد الأقراص (الشهر) |

***If answer to 77.2.3 is in short courses*** — ***إذا كانت الإجابة على 77.2.3 هى "خلال فترات قصيرة"***

| | | |
|---|---|---|
| 77.2.5 number of courses | | 77.2.5 عدد الفترات |
| 77.2.6 tablets per day | | 77.2.6 الأقراص فى اليوم |
| 77.2.7 average number of days per month | | 77.2.7 متوسط عدد الأيام فى الشهر |
| | | |

***If answer to 77.2.3 is continuously*** — ***إذا كانت الإجابة على 77.2.3 هى "باستمرار"***

77.2.8 tablets per day — 77.2.8 الأقراص فى اليوم

| | | |
|---|---|---|
| Number of tablets (day) | | عدد الأقراص (اليوم) |

77.3.1 If “YES” used to Q77.3 (oral steroids), then which one? SHOW CARDS

77.3.1 إذا كانت الإجابة "نعم" على Q77.3 (الستروید التى یتم تناولها عن طريق الفم ) أي من التالي؟ اعرض البطاقات

| | | |
|---|---|---|
| prednisolone | 1 | بريدنيزولون |
| wysolone | 2 | ويسولون |
| omnacortil | 3 | اومناكورتيل |
| wysone | 4 | ويسون |
| Medrol | 5 | ميدرول |
| Depotex | 6 | ديبوتكس |
| Other | 7 | أخرى |
| Other | 8 | أخرى |

77.3.2 what dose of tablet

77.3.2 ما هى جرعة الأقراص؟

| | | |
|---|---|---|
| Dose | | الجرعة |

77.3.3. In the last 3 months, how have you used them: TICK ONE BOX ONLY

77.3.3 كيف استخدمتهم خلال الثلاثة أشهر الماضية: ضع علامة واحدة على مربع واحد فقط

| | | |
|---|---|---|
| a) when needed | 1 | a) عند الحاجة |
| b) in short courses | 2 | b) خلال فترات قصيرة |
| c) continuously | 3 | c) باستمرار |
| d) not at all ( Go to 77.4.1) | 4 | d) لا استخدمهم على الإطلاق (انتقل إلى 77.4.1) |

**If answer to 77.3.3 is when needed:**

**إذا كانت الإجابة على 77.3.3 هى "عند الحاجة إليها": سيكون**

77.3.4 number of tablets per month

77.3.4 عدد الأقراص فى الشهر

| | | |
|---|---|---|
| Number of tablets (month) | | عدد الأقراص (الشهر) |

***<u>If answer to 77.3.3 is in short courses</u>***

***<u>إذا كانت الإجابة على 77.3.3 هى "خلال فترات قصيرة"</u>***

| | | |
|---|---|---|
| 77.3.5 number of courses | | 77.3.5 عدد الفترات |
| 77.3.6 tablets per day | | 77.3.6 الأقراص فى اليوم |
| 77.3.7 average number of days per month | | 77.3.7 متوسط عدد الأيام فى الشهر |

***<u>If answer to 77.3.3 is continuously</u>***

***<u>إذا كانت الإجابة على 77.3.3 هى" باستمرار"</u>***

77.3.8 tablets per day

77.3.8 الأقراص فى اليوم

| | | |
|---|---|---|
| Number of tablets (day) | | عدد الأقراص (اليوم) |

77.3.9. Have you used them in the last 3 months?

77.3.9 هل استخدمتهم خلال الثلاثة أشهر الماضية؟

| | | |
|---|---|---|
| Yes | 1 | نعم |
| No | 2 | لا |

77.4.1 If "YES" used to Q77.4 (oral leukotrienes), then which one? SHOW CARDS

77.4.1 إذا كانت الإجابة "نعم" على Q77.4 (مركبات الليكوترين التى يتم تناولها عن طريق الفم) أي من التالي؟ اعرض البطاقات

| | | |
|---|---|---|
| Singulair | 1 | السينجيولير |
| Monti | 2 | مونتي |
| Montair | 3 | مونتير |
| Other | 4 | أخرى |
| Other | 5 | أخرى |

77.4.2 what dose of tablet

77.4.2 ما هى جرعة الأقراص؟

| | | |
|---|---|---|
| Dose | | الجرعة |

77.4.3. In the last 3 months, how have you used them: TICK ONE BOX ONLY

77.4.3 كيف استخدمتهم خلال الثلاثة أشهر الماضية: ضع علامة واحدة على مربع واحد فقط

| | | |
|---|---|---|
| a) when needed | 1 | a) عند الحاجة |
| b) in short courses | 2 | b) خلال فترات قصيرة |
| c) continuously | 3 | c) باستمرار |
| d) not at all ( Go to 77.5) | 4 | d) لا استخدمهم على الإطلاق (انتقل إلى 77.5) |

**If answer to 77.4.3 is when needed:**

**إذا كانت الإجابة على 77.4.3 هى "عند الحاجة إليها":**

77.4.4 number of tablets per month

سيكون 77.4.4 عدد الأقراص فى الشهر

| | | |
|---|---|---|
| Number of tablets (month) | | عدد الأقراص (الشهر) |

***If answer to 77.4.3 is in short courses***

***إذا كانت الإجابة على 77.4.3 هى "خلال فترات قصيرة"***

| | | |
|---|---|---|
| **Q77.4.5**<br>number of courses | | Q 77.4.5<br>عدد الفترات |
| Q77.4.6 | | Q 77.4.6 |

| | | |
|---|---|---|
| tablets per day | | الأقراص فى اليوم |
| Q77.4.7<br>average number of days per month | | Q 77.4.7<br>متوسط عدد الأيام فى الشهر |

***If answer to 77.4.3 is continuously*** **إذا كانت الإجابة على 77.4.3 هى باستمرار**

77.4.8 tablets per day

77.4.8 الأقراص فى اليوم

| | | |
|---|---|---|
| Number of tablets (day) | | عدد الأقراص (اليوم) |

77.5.1If "YES" used to Q77.5 (ketotifen), then which one? SHOW CARDS

77.5.1 إذا كانت الإجابة "نعم" على Q77.5 (كيتوتيفن) أي من التالي؟ اعرض البطاقات

| | | |
|---|---|---|
| Ketasma | 1 | كيتاسما |
| Zaditen | 2 | زاديتين |
| Other | 3 | أخرى |
| Other | 4 | أخرى |

77.5.2 what dose of tablet

77.5.2 ما هى جرعة الأقراص؟

| | | |
|---|---|---|
| Dose | | الجرعة |

77.5.3. In the last 3 months, how have you used them: TICK ONE BOX ONLY

77.5.3 كيف استخدمتهم خلال الثلاثة أشهر الماضية: ضع علامة واحدة على مربع واحد فقط

| | | |
|---|---|---|
| a) when needed | 1 | a) عند الحاجة |
| b) in short courses | 2 | b) خلال فترات قصيرة |
| c) continuously | 3 | c) باستمرار |
| d) not at all ( Go to 78) | 4 | d) لا استخدمهم على الإطلاق (انتقل إلى Q 78) |

**If answer to 77.5.3 is when needed:** **إذا كانت الإجابة على 77.5.3 هى "عند الحاجة إليها":**

77.5.4 number of tablets per month

سيكون 77.5.4 عدد الأقراص فى الشهر

| | | |
|---|---|---|
| Number of tablets (month) | | عدد الأقراص (الشهر) |

***If answer to 77.5.3 is in short courses*** ***إذا كانت الإجابة على 77.5.3 هى "خلال فترات قصيرة"***

| | | |
|---|---|---|
| **Q 77.5.5** | | **Q 77.5.5**<br>عدد الفترات |

| | | |
|---|---|---|
| number of courses | | |
| **Q 77.5.6**<br><br>tablets per day | | **Q 77.5.6**<br>الأقراص فى اليوم |
| **Q 77.5.7**<br><br>average number of days per month | | Q **77.5.7**<br>متوسط عدد الأيام فى الشهر |

***If answer to 77.5.3 is continuously***

***إذا كانت الإجابة على 77.5.3 هى" باستمرار"***

77.5.8 tablets per day

77.5.8 الأقراص فى اليوم

| | | |
|---|---|---|
| Number of tablets (day) | | عدد الأقراص (اليوم) |

---

78. Have you had any other **injections/ epinephrine** to help your breathing at any time in the last ***12 months***?

78. هل أخذت **أبينيفرين/ أو تم حقنك بمواد أخرى** من أجل المساعدة على التنفس فى أى وقت خلال **الاثنى عشر شهراً** الماضية؟

| | | |
|---|---|---|
| Yes ( Go to Q78.1) | 1 | نعم (انتقل إلى Q 78.1) |
| No ( Go to Q79) | 2 | لا (انتقل إلى Q 79) |

78.1. What injections?

78.1. ما هى المواد الأخرى التى تم حقنك بها؟

| | | |
|---|---|---|
| Subcutaneous adrenoreceptor agonist (self-administered) | 1 | محفزلمستقبل الإدرنالين (يحقنها المريض لنفسه) |
| Long-acting or depot steroid | 2 | استرويد طويل المفعول |
| Methylxanthines | 3 | الميثيل زانثينات |
| Other | 4 | أخرى |
| Other | 5 | أخرى |
| Not known | 6 | غير معروف |

79. Have you had any suppositories to help your breathing at any time in the last 12 months?

79. هل أخذت أى تحميلة (قمع) فى أى وقت من قبل من أجل المساعدة على التنفس خلال الاثنى عشر شهراً الماضية؟

| | | |
|---|---|---|
| Yes ( Go to 79.1) | 1 | نعم (انتقل إلى Q 79.1) |
| No ( Go to Q 80) | 2 | لا (انتقل إلى Q 80) |

79.1 What suppositories? 79.1 ما هى هذه التحميلات (الأقماع) ؟

| | | |
|---|---|---|
| Aminophylline (Phyllocontin/ Truphylline/ Minomal R/ SR) | 1 | أمينوفيللين (فيلوكونتين/ تروفلين/ مينومالR / SR) |
| Theophylline | 2 | ثيوفيللين |
| Other | 3 | أخرى |
| Other | 4 | أخرى |
| Not known | 5 | غير معروف |

80 . Have you used any other remedies to help your breathing at any time in the last 12 months?

80. هل استخدمت أى أنواع أخرى من العلاج من أجل مساعدتك على التنفس فى أى وقت خلال الاثنى عشر شهراً الماضية؟

| | | |
|---|---|---|
| Yes ( Go to Q 80.1) | 1 | نعم (انتقل إلى Q 80.1) |
| No ( Go to Q81) | 2 | لا (انتقل إلى Q 81) |

80.1. What remedies? 80.1. ما هو هذا العلاج؟

| | | |
|---|---|---|
| Hypnotherapy | 1 | المعالجة بالتنويم المغناطيسي |
| Acupuncture | 2 | الوخز بالأبر |
| Homeotherapy (herbal remedies) | 3 | الطب البديل |
| Diet control | 4 | حِمية غذائية |
| Breathing exercises | 5 | تمارين تنفس |
| Swimming/ other exercises | 6 | السباحة/ تمارين أخرى |
| Reflexology | 7 | الريفلوكسولوجي (العلاج الانعكاسى) |
| Not known | 8 | غير معروف |

81. Has your doctor ever prescribed medicines, including inhalers, for your breathing?

81. هل وصف لك طبيبك أدوية تتضمن أدوية للإستنشاق ، من أجل التنفس؟

| | | |
|---|---|---|
| Yes ( Go to 81.1) | 1 | نعم (انتقل إلى Q 81.1) |
| No ( Go to Q82) | 2 | لا (انتقل إلى Q 82) |

81.1 If you are prescribed medicines for your breathing, do you normally take

81.1 إذا تم وصف أدوية لك من أجل التنفس، هل عادة ما تأخذ

TICK ONE BOX ONLY

ضع علامة واحدة على مربع واحد فقط

| | | |
|---|---|---|
| a) all of the medicine? | 1 | a) جميع الأدوية؟ |
| b) most of the medicine? | 2 | b) معظم الأدوية؟ |

| | | |
|---|---|---|
| c) some of the medicine? | 3 | c) بعض الأدوية؟ |
| d) none of the medicine? | 4 | d) لا شيء من الأدوية؟ |

81.2 When your breathing gets worse, and you are prescribed medicines for your breathing, do you normally take

81.2. عندما تسوء حالة تنفسك، و قد تم وصف أدوية لك من أجل التنفس، هل عادة ما تأخذ

TICK ONE BOX ONLY

ضع علامة واحدة على مربع واحد فقط

| | | |
|---|---|---|
| a) all of the medicine? | 1 | a) جميع الأدوية؟ |
| b) most of the medicine? | 2 | b) معظم الأدوية؟ |
| c) some of the medicine? | 3 | c) بعض الأدوية؟ |
| d) none of the medicine? | 4 | d) لا شيء من الأدوية؟ |

81.3 Do you think it is bad for you to take medicines all the time to help your breathing?

81.3 هل تعتقد أنه من السيىء بالنسبة لك أن تأخذ أدوية دائما من أجل المساعدة على التنفس؟

| | | |
|---|---|---|
| Yes | 1 | نعم |
| No | 2 | لا |

81.4 Do you think you should take as much medicine as you need to get rid of all your breathing problems?

81.4 هل تعتقد أنه يجب أن تأخذ كمية الادوية التى تحتاجها من أجل التخلص من جميع مشاكل تنفسك؟

| | | |
|---|---|---|
| Yes | 1 | نعم |
| No | 2 | لا |

82. Are you given regular appointments to be seen by a doctor (or nurse) for your asthma, wheezing or shortness of breath?

82. هل لديك مواعيد منتظمة لكي يراك طبيب (أو ممرض) بسبب الأزمة،أو الأزيز، أو ضيق التنفس؟

| | | |
|---|---|---|
| Yes ( Go to Q82.1) | 1 | نعم (انتقل إلى Q 82.1) |
| No ( Go to Q88) | 2 | لا (انتقل إلى Q 88) |

82.1. Are you given regular appointments with a hospital doctor?

82.1 هل لديك ميعاد منتظم من أجل أن يراك طبيب فى مستشفى؟

| | | |
|---|---|---|
| Yes | 1 | نعم |
| No | 2 | لا |

82.2 Are you given regular appointments with your general practitioner?

82.2 هل لديك ميعاد منتظم مع طبيبك العام؟

| | | |
|---|---|---|
| Yes | 1 | نعم |
| No | 2 | لا |

82.3. Are you given regular appointments with a nurse?

82.3 هل لديك ميعاد منتظم مع ممرض؟

| | | |
|---|---|---|
| Yes | 1 | نعم |
| No | 2 | لا |

83. How many times have you visited the following because of breathing problems or shortness of breath in the last 12 months?

83. كم عدد المرات التى زرت فيها الأتي بسبب مشاكل التنفس أو ضيق فى التنفس خلال الاثنى عشر شهراً الماضية؟

| | | Number of times<br>عدد المرات | |
|---|---|---|---|
| 83.1<br>nurse | 1 | | 83.1<br>ممرض |
| 83.2<br>physiotherapist | 2 | | 83.2<br>طبيب علاج طبيعي |
| 83.3<br>Practitioner of 'alternative' medicine. If yes, please specify<br>________________ | 3 | | 83.3<br>ممارس للطب البديل.<br>إذا كانت الإجابة نعم،<br>من فضلك حدد<br>___________ |

84. Have you had any clinical or laboratory tests because of asthma wheezing or shortness of breathe in the last 12 months?

84. هل أجريت اختبارات سريرية أو مختبرية بسبب الازمة أو الأزيز أو ضيق التنفس خلال الاثنى عشر شهراً الماضية؟

| | | |
|---|---|---|
| Yes ( Go to Q84.1) | 1 | نعم (انتقل إلى Q84.1) |
| No ( Go to Q 85) | 2 | لا (انتقل إلى Q85) |

84.1 How many times have you had the following in the last 12 months?

84.1 كم عدد المرات التى أجريت فيها الأتى خلال الاثنى عشر شهراً الماضية؟

| | | Number of times عدد المرات | | |
|---|---|---|---|---|
| 84.1.1 Breathing test in a laboratory specifically for lung function measures | 84.1.1.1 Spirometry (Image Show card) | | 84.1.1.1 Spirometer (الصورة اعرض البطاقة) | 84.1.1 اختبار تنفس فى مختبر خصيصاً من أجل قياس وظائف الرئة |
| | 84.1.1.2 Peak Flow Meter | | 84.1.1.2 مقياس ذروة التدفق peak flow meter | |
| | 84.1.1.3 Other, please specify | | 84.1.1.3 أخرى، من فضلك حدد | |
| 84.1.2 Skin test for allergy | | | | 84.1.2 اختبار جلدى من أجل الحساسية |
| 84.1.3 Blood test for allergy | | | | 84.1.3 اختبار دم من أجل الحساسية |
| 84.1.4 x-rays | | | | 84.1.4 أشعة إكس x-rays |

85. Are you currently working?

85. هل تعمل حاليا؟

| | | |
|---|---|---|
| Yes ( Go to Q85.1) | 1 | نعم (انتقل إلى Q85.1) |
| No( Go to 85.2) | 2 | لا (انتقل إلى Q85.2) |

85.1. How many days of work have you lost because of asthma, shortness of breath or wheezing in the last 12 months?

85.1 ما هو عدد الأيام التي لم تستطع العمل فيها بسبب الأزمة، أو ضيق التنفس أو الأزيز خلال الاثنى عشر شهراً الماضية؟

| | | |
|---|---|---|
| Number of work days lost | | عدد الأيام التى لم تعمل فيها |

85.2. Were you forced to give up working because of asthma, wheezing or shortness of breath in the last 12 months?

85.2. هل اضطررت إلى ترك العمل بسبب الأزمة، أو الأزيز أو ضيق التنفس خلال الاثنى عشر شهراً الماضية؟

| | | |
|---|---|---|
| Yes ( Go to 85.2.1) | 1 | نعم (انتقل إلى Q85.2.1) |
| No ( Go to 86) | 2 | لا (انتقل إلى Q86) |

85.2.1. When? — 85.2.1 متى؟

| | | |
|---|---|---|
| Date (ddmmyyyy) | | التاريخ (يوم شهر عام) |

86. Have there been any days when you have had to give up activities other than work (e.g. looking after children, the house, studying) because of your asthma, wheezing or shortness of breath in the last 12 months?

86. هل اضطررت من قبل إلى التوقف عن ممارسة أنشطة أخرى غير العمل (مثل العناية بالأطفال، الاهتمام بأمور المنزل، المذاكرة) بسبب شعورك بالأزمة، أو الأزيز أو ضيق فى التنفس خلال الإثنى عشر شهراً الماضية؟

| | | |
|---|---|---|
| Yes | 1 | نعم |
| No | 2 | لا |

IF NO YOU HAVE FINISHED THE QUESTIONNAIRE IF YES

إذا كانت الإجابة لا تكون قد انتهيت من الاستمارة، و إذا كانت نعم:

86.1. How many days on average each month?

86.1 ما هو متوسط عدد الأيام فى الشهر؟

| | | |
|---|---|---|
| Average number of days | | متوسط عدد الأيام |

Gender — النوع

| | | |
|---|---|---|
| Male | 1 | ذكر |
| Female | 2 | أنثى |

**Date of Birth** — **تاريخ الميلاد**

| | | |
|---|---|---|
| Date (ddmmyy) | | التاريخ (اليوم الشهر العام) |

INTERVIEW TYPE?
TICK ONE BOX ONLY

نوع المقابلة؟
ضع علامة واحدة على مربع واحد فقط

| | | |
|---|---|---|
| a) At centre face to face | 1 | a) فى مركز وجهاً لوجه |
| b) At home face to face | 2 | b) فى منزل وجهاً لوجه |
| c) By telephone | 3 | c) عن طريق الهاتف |
| d) Self completed at home | 4 | d) ملئها المجيب بنفسه فى المنزل |

**Thanks and Finished**
